# Apple Pomace as a Source of Valuable Phenolics: From Drying Kinetics to Optimization of Ultrasound-Assisted Extraction Using Conventional and Alternative Solvents

**DOI:** 10.3390/antiox15040429

**Published:** 2026-03-29

**Authors:** Silviu Măntăilă, Nicoleta Balan, Ștefania Adelina Milea, Oana Viorela Nistor, Doina Georgeta Andronoiu, Gabriel Dănuț Mocanu, Gabriela Râpeanu, Nicoleta Stănciuc

**Affiliations:** 1 Faculty of Food Science and Engineering, Dunărea de Jos University of Galati, 800201 Galați, Romania; silviu.mantaila@ugal.ro (S.M.); nicoleta.balan@ugal.ro (N.B.); oana.nistor@ugal.ro (O.V.N.); georgeta.andronoiu@ugal.ro (D.G.A.); danut.mocanu@ugal.ro (G.D.M.); gabriela.rapeanu@ugal.ro (G.R.); 2 REXDAN Research Center, Dunărea de Jos University of Galati, 800008 Galați, Romania; adelina.milea@ugal.ro

**Keywords:** apple pomace, drying, NaDES, extraction, optimization, polyphenols

## Abstract

Industrial processing of apple to obtain products like juice or cider generates a significant amount of pomace, which represents 25–30% of the fresh fruit mass. Different technologies are needed to valorize apple pomace (AP), considering its significant amount of high-value compounds, such as fiber, vitamins, and polyphenols. Hot-air convection (CA) and infrared (IR) drying are widely used methods for preserving polyphenols from by-products, such as apple pomace (AP), while also extending their shelf life. This study aimed to evaluate the influence of CA and IR drying on drying kinetics, color parameters, and the preservation of polyphenolic compounds, as well as to identify a sustainable extraction approach. Both drying methods significantly affected the color characteristics and content of polyphenols with high antioxidant activity. A significant impact was noticed at higher temperatures, which may be associated with the partial inactivation of browning enzymes. IR drying resulted in a shorter drying time and lower specific energy consumption compared to CA. Furthermore, the assessment of solvent efficiency in ultrasound-assisted extraction (UAE) indicated that the natural deep eutectic solvent (NaDES) composed of choline chloride and glycerol (1:1 molar ratio) provided superior recovery of phenolic compounds with high antioxidant activity compared to conventional solvents and the other NaDES analyzed. Optimization of UAE conditions using this polyol-based NaDES allowed for achieving an extract characterized by a polyphenolic profile dominated by flavan-3-ols (catechin and epigallocatechin), followed by phenolic acids, mainly chlorogenic acid. These results confirm the potential of AP as a valuable source of bioactive compounds and of polyol-based NaDESs as a sustainable and efficient alternative for their recovery.

## 1. Introduction

In 2024, the European Union accounted for approximately 13% of global apple production [1], generating substantial quantities of by-products from industrial processing. Apple pomace (AP), which represents about 25–30% of the fresh fruit mass, is one of the main residues. The development of effective valorization strategies for this by-product has become increasingly important due to its economic and environmental concern [2,3,4,5]. The major concern related to the large amounts of AP is its high susceptibility to microbial growth due to its moisture content (70–85%), which can lead to the degradation of bioactive compounds and environmental risk if improperly managed. To ensure storage stability and prevent spoilage, the moisture content must be rapidly reduced to around 10% [6,7,8]. Given the considerable costs associated with disposal, the identification of value-added applications for AP has become an active area of research. Currently, AP is mainly utilized as animal feed, as a source of dietary fiber and polyphenols, an ingredient in dietary supplements, or as a functional component in food formulations [2,3]. Once dehydrated, its nutritional and functional properties make AP a promising raw material for the development of functional foods, food additives and nutraceutical products intended for human consumption [4,5]. Beyond extending its shelf life and reducing storage volume, it is essential to identify accessible and efficient drying techniques that preserve the bioactive compounds present in the plant matrix [8].

Various drying techniques applied on AP have been reported in the literature, including hot-air convection (CA) [7,9], infrared (IR) drying [8], microwave drying [10], hybrid systems [8,10], and freeze drying [11,12]. Microwave-assisted drying enables rapid moisture removal. However, it presents limitations like non-uniform heating, which can cause localized overheating, resulting in lower product quality and inconsistent temperature profiles, and the need for high-capacity processing equipment, especially for processing at high-power levels [13]. Freeze drying is widely recognized for its superior ability to preserve bioactive compounds, but the industrial applications are limited by the high capital and operational costs, as well as its substantial energy consumption [14].

Nowadays, there is an increase in consumer demand for safe foods of high nutritional and functional quality. This trend triggers the need for economically efficient drying methods and equipment implemented at the industrial level that are able to offer the advantage of preserving the nutrients and bioactive principles of the food in their native state. In this context, CA and IR drying were selected in the present study due to their relatively low cost, suitability for industrial application, and potential to combine energy efficiency with the advantage of preserving the polyphenolic compounds. Although both methods have been previously studied [7,8,9], most reports address them separately, and comparative evaluations under similar conditions, particularly for AP, remain limited.

To recover polyphenolic compounds from AP, various extraction techniques have been explored, including conventional maceration and ultrasound-assisted extraction (UAE) using solvents such as methanol, ethanol, acetone, water, or their mixtures [15,16]. In recent years, increasing attention has been directed toward the integration of green extraction technologies, particularly UAE combined with natural deep eutectic solvents, NaDESs [11,16].

NaDESs have attracted growing interest due to their favorable properties, including thermal and chemical stability, non-flammability, low volatility, low cost, and environmental sustainability. Moreover, they exhibit a high capacity for solubilizing a wide range of organic compounds [12,15]. NaDESs are typically composed of naturally derived components, such as organic acids, sugars, or amino acids, formed through hydrogen bonding interactions between a hydrogen bond acceptor (HBA) and a hydrogen bond donor (HBD) [11]. Rashid et al. [16] reported that UAE combined with several NaDESs, such as choline chloride:glycerol (1:2), choline chloride:lactic acid (1:3), and choline chloride:citric acid (1:1) with 30% water addition, provided superior efficiency in extracting polyphenolic compounds from AP, exhibiting enhanced antioxidant and lipid peroxidation inhibitory activity compared to 70% (*v*/*v*) ethanol. However, despite these findings, the optimization of the extraction condition using a NaDES system based on choline chloride and glycerol at a 1:1 ratio remains insufficiently explored. The novelty of the present study resides in the systematic investigation of various molar ratios of NaDES systems, with particular emphasis on the influence of their physicochemical properties (such as tunability and viscosity) on mass transfer phenomena and, consequently, on the extraction efficiency of polyphenolic compounds from AP. Furthermore, special attention is paid to the affinity of phenolic compounds for NaDES solvents, which is expected to differ significantly from that observed in conventional solvents such as ethanol, potentially leading to enhanced solubilization and more selective extraction of target bioactive compounds.

The main objective of this study was to evaluate the impact of two drying methods on drying kinetics, color parameters, and the preservation of bioactive compounds from AP. Additionally, the study aimed to optimize and validate UAE conditions using NaDES as a sustainable and efficient approach for the recovery of valuable bioactive compounds.

## 2. Materials and Methods

### 2.1. Plant Materials

Fresh Crimson Snow apples were purchased from a local store in Galați, Romania, in September 2025. Fruits at full maturity, with 15.31 ± 0.33 °Brix (measured using a Digital Refractometer ORM 1RS, Kern & Sohn GmbH, Balingen, Germany), selected based on uniform size and color and lack of visible defects, were stored at 4 ± 1 °C until processing into AP. They were first washed with distilled water and gently dried with paper towels to eliminate any excess moisture. The apples were then quartered and processed using a fruit and vegetable juicer with an auger system (Biovita SJ5000, 240 W, stainless steel, Galați, Romania) to extract the juice, resulting in the separation of the AP.

### 2.2. Reagents

Folin–Ciocâlteu reagent, sodium carbonate, DPPH (2,2-diphenyl-1-picrylhydrazyl), aluminum chloride, ethanol (≥96%), choline chloride, lactic acid, and glycerol were purchased from Sigma-Aldrich (Steinheim, Germany). Methanol (≥99.9%), formic acid (≥98%), and analytical-grade HPLC standard compounds, including phenolic acids (gallic, 4-hydroxybenzoic, protocatechuic, vanillic, ellagic, syringic, caffeic, *p*-coumaric, ferulic, sinapic, and chlorogenic acids), flavan-3-ols (epigallocatechin, catechin, epicatechin, and epicatechin gallate), flavonols (quercetin 3-diglucoside, quercetin 3-glucoside, quercetin, quercetin dihydrate, and isorhamnetin), flavones (apigenin and luteolin), anthocyanins (peonidin 3-*O*-glucoside and cyanidin 3-*O*-glucoside), and flavanones (hesperidin), were obtained from Sigma-Aldrich (Steinheim, Germany).

### 2.3. Drying Methods Applied to Fresh Apple Pomace

The obtained AP (DW of 33.06 ± 0.77%) was subjected to drying treatments at three different temperatures (50 °C, 60 °C, and 70 °C) using CA drying with a Hendi Profi Line food dehydrator of 1000 W (Brașov, Romania) and IR drying with a Concept SO4000 Infra dehydrator of 500 W (Chocen, Czech Republic). For CA drying, the air velocity was 1.2 ± 0.1 m/s (measured with a hotwire thermo-anemometer VT 115 Kimo Instruments, Millgrove, ON, Canada), and the relative humidity was 13.1% ± 0.8%, measured with a thermo-hygrometer (EE33 Series) equipped with a sensing probe (E+E Elektronik Ges.m.b.H., Engerwitzdorf, Austria). The drying temperatures and the thickness of the material were selected based on data reported in the relevant scientific literature [7,8].

Thus, 30 ± 1 g of AP, with a general thickness of 0.75 ± 0.01 cm (measured with a digital caliper IP54, Wuppertal, Germany), were subjected to drying. The sample mass (measured with a Precisa EP–125SM digital scale, Iași, Romania) was recorded at 30 min intervals until three consecutive equal values were obtained, which was considered the equilibrium point.

After drying, the AP was ground with an electric grinder (coffee grinder, Heinner HCG-150SS; Heinner, Network One Distribution, Bucharest, Romania) and stored in plastic bags in a dark place to prevent degradation.

### 2.4. Analysis of Drying Kinetic Parameters of Apple Pomace

The obtained data were used to calculate the moisture ratio (MR), according to Equation (1), and the drying rate (DR), according to Equation (2). Subsequently, graphical representations were generated to show the evolution of the moisture content of AP over time for CA and IR, respectively, as well as the corresponding drying rate [7,17]:

(1) $$MR\;\left(\mathrm{dimensionless}\right)=\frac{M_{AP_{t}}-M_{AP_{e}}}{M_{AP_{0}}-M_{AP_{e}}}$$

where $M_{AP_{t}}$ is the MR of AP at time t (kg/kg), $M_{AP_{e}}$ is the MR of AP at the equilibrium point (kg/kg), and $M_{AP_{0}}$ is the initial MR of AP (kg/kg).

(2) $$DR=\frac{M_{AP_{t+\Delta t}}-M_{AP_{t}}}{30}$$

where $M_{AP_{t+\Delta t}}$ is the MR of AP at time *t* + ∆*t*, while 30 represents the time interval between two consecutive measurements (min).

Equation (3), which is based on Fick’s second law of diffusion, was used to determine the effective moisture diffusion (D_eff_) of AP at different drying regimes tested for both drying techniques:

(3) $$MR=\frac{8}{\pi^{2}}\overset{\infty }{\underset{n=0}{\sum }}\frac{1}{{\left(2n+1\right)}^{2}}exp\left[-{\left(2n+1\right)}^{2}\times \frac{\pi^{2}\left(D_{eff}\times t\right)}{4L^{2}}\right]$$

where MR is the moisture ratio of the AP samples, t is the time (s), L is the thickness of the material (m), and D_eff_ is the effective moisture diffusion (m^2^/s).

The D_eff_ values calculated using the slope from the regression equation resulted from plotting the logarithmic MR values as a function of time for each drying temperature, which were subsequently used to estimate the activation energy (E_a_). The graphical representation of the logarithmic D_eff_ values as a function of the inverse of the absolute temperature (1/K) allowed for calculating the E_a_ using the Arrhenius equation (Equation (4)):

(4) $$D_{eff}=D_{0}\exp\left(-\frac{E_{a}}{R\times T}\right)$$

where D_0_ is the pre-exponential factor of the Arrhenius equation (m^2^/s), E_a_ is the activation energy (kJ/mol), T is the absolute temperature (K), and R is the universal gas constant (R = 8.31451 J/ (mol · K) [8,17].

### 2.5. Fitting Data to Drying Curves of Mathematical Models

The experimental results obtained for the CA and IR drying of plant material (AP) at the three levels of temperatures were fitted to seven commonly used mathematical models (Table S1, Supplementary Materials) [9,18,19,20,21,22,23,24].

For each evaluated model, the specific coefficients (k, n, a, b, and c) were determined. The statistical parameters root mean square error (RMSE), reduced chi-square (χ^2^), and the coefficient of determination (R^2^) were used as selection criteria to assess the goodness of fit of each model to the drying curves for both drying techniques.

### 2.6. Powder Color Parameters in AP

Fresh AP (AP_F) was used as the control sample, while the dried AP powders were coded according to the drying temperatures (50, 60, and 70 °C) and the applied techniques (CA or IR). The color parameters were measured using an NR110 precision colorimeter (Shenzhen 3nh Technology Co., Guangdong, China), reporting the values of the color parameters: L* (where 100 is white and 0 is black), a* (where 60 to 0 is green and 0 to 60 is red intensity), b* (where 60 to 0 is blue intensity and 0 to 60 is yellow intensity), C (chroma or color intensity), and h (hue angle).

Total color change (ΔE) and browning index (BI) were used as indicators of color changes produced by the drying technique and processing temperatures, calculated according to the formulas reported by Ershadfarkar et al. [25].

(5) $$\Delta E={\left[{\left(L_{0}^{*}-L^{*}\right)}^{2}+{\left(a_{0}^{*}-a^{*}\right)}^{2}+{\left(b_{0}^{*}-b^{*}\right)}^{2}\;\right]}^{\frac{1}{2}}$$

(6) $$\mathrm{Browing}\;\mathrm{Index}\;\left(\mathrm{BI}\right)=\frac{100\left[\left(\frac{a^{*}+1.75L^{*}}{5.645L^{*}+a^{*}-3.012b^{*}}\right)-0.31\right]}{0.17}$$

Correspondingly, the values for the yellowness index (YI) and whiteness index (WI) were also calculated based on Equations (7) and (8) [17]:

(7) $$\mathrm{Yellowness}\;\mathrm{Index}\;\left(\mathrm{YI}\right)=\frac{142.86b^{*}}{L^{*}}$$

(8) $$\mathrm{Whiteness}\;\mathrm{Index}\;\left(\mathrm{WI}\right)=100-\sqrt{\;{\left(100-L^{*}\right)}^{2}+{\left(a^{*}\right)}^{2}+{\left(b^{*}\right)}^{2}}$$

where $L_{0}^{*}$, $a_{0}^{*}$, and $b_{0}^{*}$ are the color parameters for the control (AP_F); L*, a*, and b* are the color parameters of dried AP powders: L* (lightness/darkness), a* (redness), and b* (yellowness).

### 2.7. Extraction of Bioactive Compounds from Apple Pomace Using Ultrasound-Assisted Technique

To recover the bioactive compounds with high antioxidant activity from AP, UAE was employed using two classes of solvents: conventional and alternative. For each extraction, 1 g of AP and 10 mL of solvent were subjected to sonication (Digital Ultrasonic Bath Mod. DU-32; 131 ARGOLAB; Capri, Italy) at 50 °C for 30 min, keeping the power (100 W) and amplitude (40 kHz) of the ultrasound bath constant [15,16,26]. The solvents used for the assay were distilled water and 80% (*v*/*v*) ethanol as classical solvents, and the alternative solvents (NaDESs) with acidic solvents were choline chloride:lactic acid (ChCl:La) in a molar ratio of 1:1 and 1:2, respectively, and a neutral one of choline chloride:glycerol (ChCl:G) in a molar ratio of 1:1 and 1:2, respectively. Each NaDES was supplemented with 20% water to reduce viscosity and surface tension [11]. After sonication, the mixtures were centrifugated using a Universal 320R centrifuge (Hettich, Tuttlingen, Germany) at 6000× *g* for 10 min at 4 °C. The resulting supernatants were carefully collected for subsequent analyses [27].

### 2.8. Polyphenolic Profile and Antioxidant Activity of Apple Pomace Powders

The total anthocyanins content (TAC), total polyphenol content (TPC), Total Flavonoid Content (TFC), and antioxidant activity of the AP extracts were quantified using established methods. TAC was quantified using the pH differential method described by Balan et al. [27], and the results were expressed as milligrams of cyanidin-3-glucoside per gram of dried AP (mg C3G/g DW). For TPC, the procedure of Serea et al. [28] was followed, where the absorbance of the samples was measured at 765 nm using a Biochrom Libra 22 UV/Visible Spectrophotometer (177 Holliston, MA, USA). The results were calculated using the calibration curve (y = 0.8831x + 0.0084, R^2^ = 0.9986), which was linear in the range of 0–0.5 mg gallic acid/mL and expressed as milligrams of gallic acid equivalents per gram of dried AP (mg GAE/DW).

For TFC, the method outlined by Mërtiri et al. [29] was applied. Absorbance readings were recorded at 440 nm, and the flavonoid content was determined by comparing the sample values to a calibration curve (y = 8.7133x − 0.1041, R^2^ = 0.9948), which was linear in the range of 0–0.35 mg quercetin/mL and expressed as milligrams of quercetin equivalents per gram of dried AP (mg QE/DW).

Antioxidant activity was measured using the DPPH method, as described by Serea et al. [28]. Absorbance was measured at 515 nm, and the antioxidant capacity was calculated by subtracting the absorbance of the control from that of the extract. The results were expressed as millimoles of Trolox equivalents per gram of dry weight (mmol TE/g DW) based on a pre-established calibration curve (y = 0.492x − 0.0056, R^2^ = 0.9974, which was linear in the range of 0–2 mmol Trolox/mL).

### 2.9. High-Performance Liquid Chromatography

The analysis of polyphenolic compounds was conducted using High-Performance Liquid Chromatography (HPLC), as outlined by Balan et al. [27], at detection wavelengths of 280 nm, 320 nm, and 520 nm. An Agilent 1200 HPLC system (Agilent Technologies, Santa Clara, CA, USA) was employed for the analysis, which included an integrated degasser, a quaternary pump, a chromatographic column, and a diode-array detector (DAD). Data acquisition and processing were automated using Agilent ChemStation software (Rev. B.04.03), with calibration curves derived from analytical standards.

The polyphenolic compounds were separated using a BDS Hypersil C18 column (Thermo Fisher Scientific, 1228 Titan Way, Sunnyvale, CA, USA) (150 × 4.6 mm, 5 µm) maintained at 30 °C. A 15 µL injection volume was used, and the mobile phase flow rate was set to 1 mL/min. The mobile phase consisted of 100% methanol (solvent A) and 10% formic acid (solvent B), with the following gradient program: 0–20 min, 9% A/91% B; 20–30 min, 35% A/65% B; 30–40 min, 50% A/50% B; and 40–45 min, 9% A/91% B. The results were quantified and expressed as mg/100 g DW of AP.

### 2.10. Design of the Experimental Plan for Optimizing the Ultrasound-Assisted Extraction of Bioactive Compounds from Apple Pomace

Polyphenolic compounds in plant matrices exhibit various affinities for NaDESs during UAE. In general, their extraction yield is influenced by solvent polarity, viscosity, and electrical conductivity, the proportion of water added to the solvent, extraction time, and temperature [15,16,26]. To determine the optimal conditions, specifically, A: temperature (°C), B: extraction time (min), and C: water content in the solvent (%), for obtaining an extract rich in polyphenols (R1: TPC, mg GAE/g DW), flavonoids (R2: TFC, mg QE/g DW), and exhibiting high antioxidant activity (R3: DPPH), the response surface methodology (RSM) approach was employed using a Box–Behnken design. Table 6 summarizes the three independent variables (A, B, and C) along with their corresponding three levels (A: 40–60 °C, B: 30–60 min, and C: 20–40%), resulting in a total of 18 experimental runs, of which six represent the intermediate level of each factor under investigation.

### 2.11. Statistical Analysis

The fitting of the drying models, including the estimation calculation of the specific coefficients (k, n, a, b, and c) and the statistical indicators RMSE, χ^2^, and R^2^ used to evaluate the goodness of fit, was performed using MATLAB R2025b (MathWorks, Carlsbad, CA, USA). The optimization and validation of the UAE condition for the recovery of the bioactive compounds from AP with selected NaDES were done with Design-Expert software, version 13.0 (Stat-Ease, Minneapolis, MN, USA).

Analysis of variance (ANOVA) was applied to evaluate the effect of experimental factors on drying kinetics, color parameters, polyphenolic profiles, antioxidant activity, and individual compounds identified by HPLC. Tukey’s test was used for post hoc comparisons at a 5% significance level. Prior to ANOVA, the assumptions of normality and homogeneity of variance were verified. Principal Component Analysis (PCA) and Pearson correlation analyses were carried out using Minitab software, version 19 (Romsym Data, Bucharest, Romania).

All analyses were performed in triplicate, and results were expressed as mean ± standard deviation (n = 3), where replicates represent independent analytical determinations.

## 3. Results and Discussion

### 3.1. Drying Curve Analysis and Mathematical Modeling

**Figure 1:**
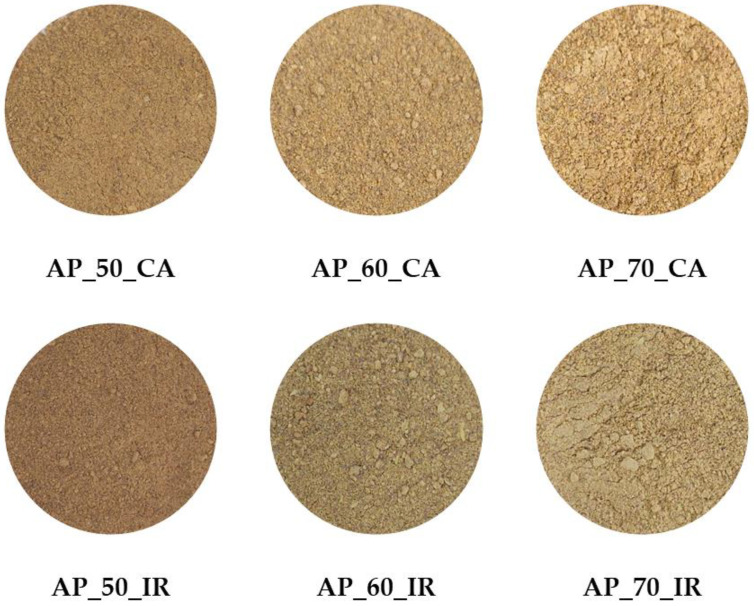
AP powders dried using convective air and infrared drying methods at different temperatures.

Fresh AP was subjected to CA and IR drying processes, studying the effect of temperature and drying technique on the plant matrix. The visual appearance of the samples after drying is shown in Figure 1.

**Figure 2:**
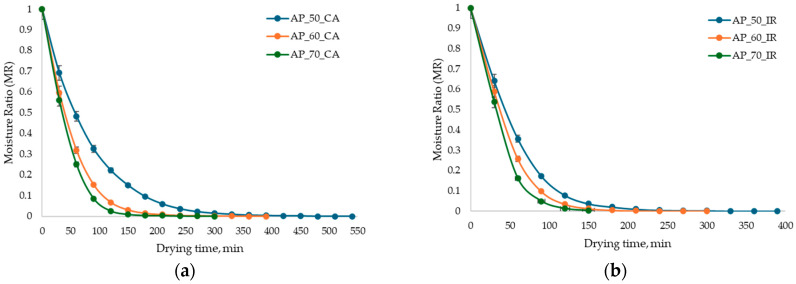
Moisture ratio versus drying time of AP under convective air (**a**) and infrared (**b**) drying methods at 50 °C, 60 °C, and 70 °C.

As shown in Figure 2, increasing the drying temperature resulted in a reduction in drying time and a decrease in the final moisture ratio (MR) of the samples for both drying methods. Specifically, the results indicate that a lower final MR, corresponding to lower residual moisture, was achieved at higher drying temperatures [17].

The drying curves (Figure 2) highlight a significant reduction (*p* < 0.05) of AP moisture content with the temperature increase by 37.50% for CA and 45.45% for IR. This behavior is consistent with the drying theory, as higher temperatures enhance the vapor pressure gradient between the material and the surrounding air, thereby accelerating moisture diffusion and evaporation. The observed reduction in drying time and final MR is attributed to the accelerated migration of water from the interior of the plant matrix to the surface, a process promoted by the increased heat transfer rate between the plant material and the heating medium within the drying chamber [7,17].

**Figure 3:**
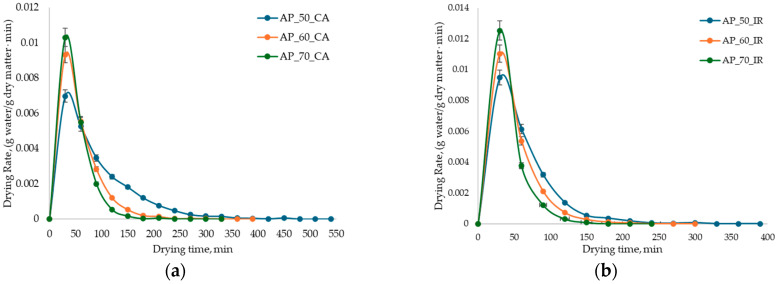
Variation in the drying rate of AP during convective (**a**) and infrared (**b**) drying at 50 °C, 60 °C, and 70 °C.

Similar results were reported by Kara & Doymaz [7], who performed the CA drying of AP at temperatures between 50 and 80 °C, as well as by Sun et al. [8], who studied the IR drying of fresh AP and heat-pretreated pomace using CA, respectively. According to Figure 2, the drying time of AP by CA varied between 360 and 540 min, while for IR, the time required to reach a constant mass was much shorter (*p* < 0.05), varying between 240 and 390 min. The DR of AP (Figure 3) exhibited two distinct phases without achieving a constant drying rate.

Moreover, the specific energy consumption decreased significantly (*p* < 0.05) with the increasing temperature from 50 to 70 °C from 22.50 ± 0.25 to 15.00 ± 0.42 kW·h/kg AP powder in the case of CA drying. In contrast, IR drying showed significantly (*p* < 0.05) lower values, ranging from 8.13 ± 0.30 to 5.00 ± 0.15 kW·h/kg AP powder. These results indicate that higher drying temperatures improve energy efficiency in both methods. Additionally, IR drying requires substantially less energy compared to CA, highlighting its potential as a more economically advantageous, energy-efficient, and industrially relevant drying technique.

The first phase (initial 30 min) involved preheating the material, during which the MR values were the highest, ranging from 0.5590 to 0.6918 g water/g DW for CA drying and from 0.5367 to 0.6406 g water/g DW for IR drying. This indicates an accelerated DR directly correlated with the increase in temperature. This rapid initial phase is attributed to the evaporation of unbound and surface water [17].

The second phase was characterized by a decline in the drying rate, reflecting the slower removal of bounded water, eventually reaching the equilibrium moisture content. These drying behaviors are closely associated with the microstructural changes induced by the drying methods. According to Sun et al. [8], CA causes significant structural alterations, including cell wall shrinkage and the formation of large intercellular spaces, which increase the porosity and specific surface area. In contrast, IR drying preserves tissue integrity, inducing only minor surface-level changes. Consequently, IR drying, due to its deeper penetration and direct conversion of radiation into thermal energy, ensures more uniform moisture removal, shorter drying times, and higher quality of the final product by preserving texture and structural integrity [29,30].

### 3.2. Fitting Drying Curves to Mathematical Models

The moisture ratio (MR) values obtained for both drying methods applied to AP at a temperature of 50 °C, 60 °C, and 70 °C were used to perform a nonlinear regression analysis of seven thin-layer drying models (Table S2, Supplementary Materials). To identify the optimal model which describes the best experimental drying curves of AP, the MR values were fitted to seven mathematical models commonly reported in the literature (Table S1, Supplementary Materials) [31]. R^2^, RMSE, and χ^2^ were the statistical indicators calculated for each characteristic equation to evaluate the goodness of fit of the drying model to the experimental drying curves.

The R^2^ ranged from 0.76907 to 0.99999, while RMSE values were between 4.74 × 10^−3^ and 9.6 × 10^−2^. The χ^2^ values were situated within an interval of 5.74 × 10^−6^ to 1.19 × 10^−2^. The Midilli model provided the best fit for the thin-layer drying data of AP, exhibiting the highest R^2^ values (0.99979–0.99999) and the lowest RMSE (4.74 × 10^−3^–1.13 × 10^−3^) and χ^2^ (5.74 × 10^−6^–1.05 × 10^−5^) values for both drying methods. Similar findings were reported by Kara & Doymaz [7], who evaluated ten mathematical models of AP drying at temperatures between 50 °C and 80 °C and identified that the Midilli model provided the best fit with the experimental results for all applied temperatures. However, as shown in Table S2 (Supplementary Materials), the Page model exhibited R^2^ values (0.99978–0.99998) comparable to those of the Midilli model. Moreover, the RMSE and χ^2^ values calculated for the Page model were also lower, ranging from 4.88 × 10^−3^ to 1.22 × 10^−3^ for RMSE and from 9.19 × 10^−6^ to 1.51 × 10^−5^ for χ^2^. The similarity was demonstrated by plotting predicted and experimental MR values for Page and Midilli models (Figure S1, Supplementary Materials). The Page model has frequently been identified as providing the best fit to experimental data for the CA drying of AP [31] and for the IR of AP pre-dried via CA [8].

### 3.3. Effective Moisture Diffusivity and Activation Energy

The effectiveness of moisture diffusivity was determined according to Equation (4), with values obtained for CA drying ranging between 1.40 ± 0.01 and 2.90 ± 0.09 × 10^−9^ m^2^/s, whereas the IR drying process yielded considerably higher values between 2.14 ± 0.02 and 3.56 ± 0.11 × 10^−9^ m^2^/s (Table 1). Wang et al. [9] reported D_eff_ values for AP dried at 75–105 °C using CA, ranging from 1.91 × 10^−9^ to 3.94 × 10^−9^ m^2^/s, while Sun et al. [8] reported D_eff_ values for IR drying at 55–75 °C, ranging from 3.48 × 10^−9^ to 6.48 × 10^−9^ m^2^/s. As shown in Table 1, the temperature increase led to a directly proportional increase in the thermal energy transferred to the AP, resulting in a faster diffusion rate of water molecules [7,17].

The D_eff_ values obtained in this study were similar to the specific values reported for drying biological materials [17,32]. IR drying produced a more efficient mass transfer than hot-air drying, and this result is in good agreement with previous studies [8,17]. According to the data shown in Table 1, with a high accuracy of experimental values, CA drying presented an activation energy (E_a_) of 33.71 ± 0.92 kJ/mol, whereas IR drying exhibited a lower value of 23.64 ± 0.92 kJ/ mol (*p* < 0.05), this effect being attributed to the accelerated rate of water migration in AP. The E_a_ value obtained for CA drying aligns well with results reported by Kara & Doymaz [7] (29.65 kJ/mol) and by Llavata et al. [4] (38.21 kJ/mol). For IR drying, the E_a_ value was lower than that reported by Sun et al. [8], which was 31.42 kJ/mol for AP and 29.78 kJ/mol for hot-air pre-dried AP. The D_eff_ and E_a_ values are significantly influenced by the type of AP used, as well as by the drying method and temperature applied for producing AP powder.

### 3.4. Color Parameters of Apple Pomace Powders

Table 2 presents the color parameters determined for AP powders obtained using the two drying methods, CA and IR, at temperatures ranging from 50 °C to 70 °C. It can be observed that the lightness (L*) value for IR-dried powders increased compared with the control and CA-dried powders.

The Pearson correlation was used to highlight the linear relationship between two variables, indicating both the strength and the direction of the correlation, with positive correlations shown in red and negative correlations in blue. Figure S2 (Supplementary Materials) revealed a strong positive relationship between the drying temperature and L* value, with the R^2^ value of both 0.977 for CA and 0.934 for IR. The lightness (L*) of AP powders increased progressively with the drying temperatures, an effect attributed to conformational changes in browning-related enzymes, like polyphenol oxidase (POD) and peroxidase (POX), which partially reduced their activity when the temperature increased up to 70 °C [33]. The highest L* values of 52.35 ± 0.60. were recorded at 70 °C.

Regarding the redness (a*) values, it was observed that powders dried at 50 °C and 60 °C by CA exhibited significantly higher values than the control. This increase can be attributed to the formation of enzymatic browning compounds, resulting from the degradation of apple cell tissues during pressing, which allows POD/POX to interact with phenolic compounds [34]. In the case of CA drying at 70 °C, a* values decreased to 13.09 ± 0.25, indicating that chromatic reactions induced by PPO/POD activity are minimized by partial thermal inactivation, which is supported by higher polyphenols content (Table 2) in the powder and by the negative correlation between a* and TPC (r = −0.675), as shown in Figure S2A (Supplementary Materials).

In contrast, IR-dried powders showed lower a* values than the fresh AP (AP_F), reflecting the more rapid heat transfer between AP and the drying agent, with a slight increase in a* values directly proportional to the drying temperature. For the yellowness (b*), the values showed an increasing trend with the increase in drying temperatures, with significantly higher b* values observed for IR-dried powders compared to CA-dried powders (Table 2). A positive correlation was found between b* and the drying temperature, with 0.810 for CA and 0.767 for IR drying, produced by polyphenols oxidation by PPO/POD or Maillard-specific compounds [34]. Pakulska et al. [35] reported, for AP powder dried at 70 °C, higher L* values (57.35 ± 0.71) and lower a* (9.22 ± 0.22) and b* (21.81 ± 0.21) values compared to those obtained in the present study (Table 2). In contrast, Aldosari [36] reported color values similar to those observed for the CA-dried sample at 60 °C in our study, with L* = 44.70 ± 0.88, a* = 14.90 ± 0.65, and b* = 28.16 ± 0.58 (Table 2).

One of the most important parameters for the visual perception of AP powder is the color difference (∆E). This parameter reflects how the drying process affects the final powder color compared with the control sample (AP_F). The powders obtained by both drying methods are presented in Figure 1, and noticeable differences in sample colors can be observed. The ∆E values for CA-dried powders ranged from 2.15 ± 0.50 to 11.81 ± 0.94, whereas IR-dried powders exhibited considerably higher values, ranging from 10.12 ± 0.76 to 14.61 ± 0.64, confirming that the drying method exerts a pronounced impact on the final product color. Furthermore, increasing the drying temperature from 50 °C to 70 °C led to an increase in the dependent variable, showing a strong positive correlation of 0.965 for CA and 0.896 for IR drying (Figure S2, Supplementary Materials).

Table 2 presents the variation in chroma (C*) and hue angle (h*), as well as of the yellowness index (YI), browning index (BI), and whiteness index (WI) for both drying methods. The CA-dried powders at 50 °C showed higher BI values than the control (Table 2), likely due to the activity of POD/POX, which are enzymes known for their high thermal stability [17]. As the temperature increased, BI and YI decreased, showing a strong negative correlation, while WI increased with a higher positive correlation for both drying methods (Figure S2, Supplementary Materials). This is likely a result of the faster drying process, which limits Maillard reaction formation and more effectively reduces the activity of enzymes responsible for enzymatic browning.

### 3.5. Phenolic Profile and Antioxidant Activity of Apple Pomace Powders

To identify the AP powder having the highest polyphenolic content and antioxidant activity, the samples were subjected to UAE at 30 °C for 30 min, using a liquid-to-solid (L/S) ratio of 10 mL/g and 70% (*v*/*v*) ethanol as the extraction solvent. As shown in Table 3, significant differences were observed in the TPC, TFC, and antioxidant activity (DPPH) values. The TPC values and antioxidant activity of extracts obtained from CA-dried powders increased with the temperature, confirming the importance of temperature for preserving bioactive compounds and minimizing their degradation. AP powder dried by CA at 70 °C exhibited the highest TPC (8.03 ± 0.39 mg GAE/g DW), with a corresponding antioxidant activity of 22.13 ± 0.51 mmol Trolox/g DW, whereas at 50 °C, the values were lower by 10.21% and 6.51%, respectively.

Based on Pearson correlation analysis (Figure S2, Supplementary Materials), the relationship between temperature, drying time, TPC, TFC, and DPPH were established. For both drying methods, bioactive compounds with high antioxidant activities were more available in the powder when AP was dried at a high temperature for a short time. This was deduced by considering the positive correlation between TPC and the temperature (0.789 for CA and 0.736 for IR) and the negative correlation between TPC and the drying time (−0.745 for CA and −0.748 for IR).

The strong positive correlation between TPC and DPPH observed for both drying methods (r = 0.896 for CA and 0.893 for IR) suggests that the phenolic compound polyphenols are the main contributors to the antioxidant activity. However, the increase in antioxidant activity may be partially attributed to the formation of Maillard reaction products [34] at higher drying temperatures.

For IR-dried powders, significantly lower values of phenolics were obtained compared to CA-dried powders, particularly at 50 °C, although the same increasing trend in TPC and DPPH values was observed with the increasing temperature (Table 3). For flavonoids, CA-dried powders showed a decrease with an increasing temperature, whereas IR-dried powders exhibited the opposite trend, indicating that the choice of drying technique influences flavonoid preservation in AP powders. The enhanced availability of flavonoids at lower temperatures and longer drying times can be explained by the reduced occurrence of thermal degradation processes. The decrease in flavonoid content at high temperatures was also reported by Heras-Ramírez et al. [37] in their analysis of the phenolic profile of unblanched AP dried at 50–80 °C. The authors reported a significant reduction in (-)-epicatechin and (+)-catechin at higher drying temperatures, attributed to epimerization reactions induced by thermal exposure.

The increasing trend for TPC can be explained by the fact that the enzymes released during the degradation of apple cell tissues (POD/POX) led to the oxidation of bioactive compounds. At 50 °C, the TPC values were expected to be much lower than at 70 °C due to prolonged exposure to oxygen and the faster oxidation reactions catalyzed by these thermally stable enzymes. At higher temperatures, partial enzyme inactivation prevents the loss of phenolic compounds. Llavata et al. [4] observed a higher phenolic value under hot-air convection drying at 40–120 °C, suggesting that drying at temperatures close to 80 °C ensure the highest yield of phenolic compounds and vitamin C.

The results indicate that the increase in TPC at 70 °C may be associated with the partial inactivation of oxidative enzymes such as PPO and POX, thereby reducing their ability to catalyze the oxidation of phenolic compounds in apple pomace (e.g., chlorogenic acid and phloridzin). In contrast, the decrease in TFC can be attributed to the thermal degradation of more heat-sensitive flavonoid compounds. At 50 °C, the limited inactivation of these enzymes allows the oxidation processes to continue, which may contribute to reduced TPC values.

Overall, the interplay between enzymatic activity and thermal degradation appears to be a determining factor in shaping the final phenolic profile of dried AP. Based on the reported results, AP_70_CA powder was selected for testing the extraction efficiency of NaDESs.

### 3.6. Evaluation of the Ultrasound-Assisted Extraction Using Conventional Versus Alternative Solvents

The next part of the study focused on the recovery of bioactive compounds using two conventional solvents (water and 80% (*v*/*v*) ethanol) and two NaDESs in different molar ratios. This part of the research aimed to evaluate the extraction efficiency from the selected AP powder using two NaDES systems, as described above. The composition of the solvents and pH values at 25 ± 1 °C are presented in Table 4.

The total anthocyanin content (TAC) ranged from 0.17 ± 0.02 to 2.64 ± 0.34 mg C3G/g DW. The use of 80% (*v*/*v*) ethanol and all NaDES systems ensured higher values of extracted bioactive compounds compared to water (Table 4), which can be attributed to the solvent pH. Extracts based on ChCl:G exhibited significantly higher TAC values compared to both 80% (*v*/*v*) ethanol and ChCl:La. Among the NaDES systems, ChCl:G (1:2) provided the highest anthocyanin yield, followed by ChCl:G (1:1) and ChCl:La (1:2). Bruna et al. [11], who evaluated six NaDESs based on ChCl (HBA) combined with different hydrogen bond donors, citric acid (Ca), lactic acid (La), oxalic acid (Oa), malic acid (Ma), urea (U), and urea:water (U:W), reported TAC values for freeze-dried organic AP (sp. *Story*) ranging from 9.68 ± 2.23 to 64.81 ± 4.65 µg malvidin-3-glucoside equivalent/mL extract. These values are considerably lower than those obtained in the present study for 80% (*v*/*v*) ethanol and the NaDES systems, which ranged from 186.76 ± 29.58 to 280.14 ± 71.69 µg C3G/mL extract.

The higher affinity of anthocyanins extracted from AP for the NaDES used in UAE has been discussed in the literature. Solvent viscosity is considered the main factor limiting the extraction efficiency due to the strength of hydrogen bonds formed between HBD and HBA components and associated intermolecular interactions [12]. Zhang et al. [37] reported that ChCl:La (1:1) was inferior to ChCl:G (1:2) for anthocyanin extraction, which was attributed to the lower viscosity of ChCl:G, showing an optimal HBD:HBA ratio for maximizing TAC at 1:2.

As shown in Table 4, TPC ranged from 3.13 ± 0.11 to 6.42 ± 0.10 mg GAE/g DW, with high differences attributed to the characteristic properties of the solvents used. For example, in case of the acid-based NaDES, increasing the molar ratio led to a 35.36% increase in TPC, explained by the high affinity of certain phenolic compounds due to lower pH and viscosity (Table 4). For the polyol-based NaDES, AP phenolic affinity was significantly higher at a 1:2 molar ratio than at 1:1, increasing the extraction yield by 34.38%.

Comparing the two types of NaDESs, ChCl:La was clearly superior to ChCl:G, which is in good agreement with the results reported by Deniz et al. [38]. However, the authors observed lower TPC values of 0.28 mg GAE/g DW and 0.63 mg GAE/g DW for AP extracted with ChCl:La (1:1 molar ratio) and ChCl:G (1:2 molar ratio), respectively, compared to the yields obtained in our study (Table 4). Wang et al. [15] reported a considerably lower yield of 2.18 mg GAE/g fresh AP for hydroalcoholic extracts (70% (*v*/*v*) ethanol). The authors also conducted a preliminary study on the extraction efficiency of several NaDESs for bioactive compounds from fresh AP, including ChCl:La at a 1:2 molar ratio with 70% water, which yielded 3 mg GAE/g fresh AP. In contrast, the use of ChCl:La in the present study resulted in a substantially higher phenolic yield of 6.42 ± 0.10 mg GAE/g AP with 20% water added. These differences are attributed to the distribution of phenolic compounds within the plant matrix, the form of the material subjected to extraction (fresh vs. dried), and specific solvent characteristics, including viscosity, pH, and electrical conductivity [39].

Rashid et al. [16] investigated seven different NaDESs for the extraction of bioactive compounds from freeze-dried AP. Under their experimental conditions (40 °C, 30 min, 30% water, and S/L ratio 30 mL/g), ChCl:G at a 1:2 molar ratio yielded a TPC of 5.6 mg GAE/g AP, a TFC of 4.5 mg QE/g AP, and an antioxidant activity of 77%. In contrast, the extract obtained from AP dried at 70 °C by CA exhibited a TPC of 3.13 ± 0.11 mg GAE/g DW, a TFC of 1.05 ± 0.02 mg QE/g DW, and an antioxidant activity of 25.44 ± 0.26 mmol Trolox/g DW (67.09 ± 0.72%).

These differences are primarily attributed to the drying method applied to the plant matrix, as freeze drying better preserves phenolic compounds compared to CD drying. ChCl:G (1:1 molar ratio) was selected for optimization because it provided higher antioxidant activity (25.44 ± 0.26 mmol Trolox/g DW) and flavonoid content (1.05 ± 0.02 mg QE/g DW) relative to the other NaDESs and conventional solvents. This phenomenon can be explained by the solvent’s pH, the affinity of the flavonoids for alcohol-based NaDESs, and the preservation of their protonated forms. Additionally, the high number of hydrogen bonds formed between the solute and the solvent compared to acid-based NaDESs enhances the extraction efficiency of the compounds with high antioxidant activity [16,40,41,42].

### 3.7. Analysis of Polyphenolic Profile of the Extracts by High-Performance Liquid Chromatography

The HPLC analysis of the hydroalcoholic extract (Table 5) identified nine phenolic compounds in quantifiable amounts, while three compounds were detected only as traces. The extraction was performed by UAE at 50 °C for 30 min, using an L/S ratio of 10 mL/g and 80% (*v*/*v*) ethanol. Among the identified compounds, flavan-3-ols predominated, accounting for 90.90% of the total polyphenolic profile.

Fariñas-Mera et al. [43] performed a conventional extraction of AP dried at 45 °C using 50% (*v*/*v*) ethanol under agitation (180 rpm) at 50 °C for 240 min, with an L/S of 20 mL/g. The authors identified fourteen polyphenolic compounds, with the extract being particularly rich in phenolic acids (80.80 mg/100 g DW), followed by dihydrochalcones (68.97 mg/100 g DW). These differences may be attributed to the variation in drying conditions, extraction technique, solvent used, and the characteristics of the analyzed plant matrix.

The exclusive use of HPLC represents a methodological limitation, as compound identification relied mainly on retention times and comparison with available standards. Coupling HPLC with mass spectrometry (HPLC–MS) would have enabled the identification and quantification of a larger number of compounds. Nevertheless, this approach was appropriate for the aim of the present study, which sought to evaluate how solvent characteristics influence the recovery of polyphenolic compounds. The physicochemical properties of acid- and polyol-based NaDESs, including density and viscosity, pH values, and their affinity for phenolic compounds determined by the similarity in polarity between the solvent and the extracted compounds, led to significant variations in both the polyphenolic profile and extraction yields. These effects reflect a differential extraction of polyphenolic compounds according to their polarity, resulting in distinct extractive profiles, which could be adequately monitored and compared using conventional HPLC.

PCA was applied to evaluate the polyphenolic profile of the five extracts (hydroalcoholic and NaDES), allowing for an investigation on the influence of solvent type on the extraction of major polyphenolic compounds. As presented in Table S3 (Supplementary Materials), the first two components (PC1 and PC2) are sufficient to explain the complexity of the polyphenolic profile of the AP extracts, accounting for 69.90% of the total variance. According to Figure S3 (Supplementary Materials), the PCA score illustrates the distribution of solvents based on their affinity for specific groups of polyphenols.

The first quadrant included ChCl:G at a 1:2 molar ratio, which exhibited the highest content of phenolic acids: gallic, sinapic, and protocatechuic acids, as well as the anthocyanin peonidin-3-*O*-glucoside.

In the second quadrant, ChCl:G at a 1:1 molar ratio was grouped with a higher number of polyphenolic compounds. For the alcohol-based NaDES, significantly higher contents of catechin, epicatechin, epicatechin gallate, gallic acid, and peonidin-3-*O*-glucoside were observed, confirming the strong affinity of these compounds for a pH close to 4.0 compared to the acid-based NaDES with a lower pH. Flavonoids have a strong affinity for alcohol-based NaDESs due to the preservation of their protonated form, which enhances their structural stability and maintains their bioactivity [39]. During UAE at 50 °C, catechins present greater stability in the ChCl:G (1:2) system than in ChCl: La (1:2) due to the higher number of hydroxyl groups, which promotes the formation of hydrogen bonds between the solvent and flavonoids, thereby facilitating the improvement of the extraction yield [40]. In the third quadrant, ChCl:La at a 1:1 molar ratio exhibits a polyphenolic profile rich in phenolic acids, explained by the lower pH value of the solvent. In the fourth quadrant, the NaDES composed of choline chloride and lactic acid at a 1:2 molar ratio, along with the hydroalcoholic solvent, showed a similar capacity to recover significant amounts of epigallocatechin and quercetin.

According to Figure S3 (Supplementary Materials), it can be observed that the hydroalcoholic and ChCl:La (1:2 molar ratio) solvents clustered together (Figure S4, Supplementary Materials), indicating a similar extraction level for epigallocatechin and quercetin. As the similarity decreased, ChCl:G (1:2) formed a distinct group (Figure S3, Supplementary Materials) situated close to the first quadrant in Figure S4 (Supplementary Materials). ChCl:G (1:1) presents a lower value of similitude in hierarchical cluster analysis, while ChCl:La (1:1) is separate from the other extracts, a pattern also noticeable in Figure S4 (Supplementary Materials).

The phenolic profiles showed reduced similarity, especially in extracts obtained using alternative solvents. The differences in extraction efficiency between acid-based NaDESs and polyol-based NaDESs for individual phenolic compounds can be attributed to several parameters, including the HBD:HBA molar ratio, pH, viscosity, polarity, and the specific affinity of each phenolic class for a given solvent system [39,40,44].

Due to their polar–hydrophilic character, phenolic acids exhibit a polarity closer to that of acid-based NaDESs than to polyol-based NaDESs, which are generally less polar. This polarity matching explains the predominance of phenolic acids extracted using ChCl:La, as it promotes stronger hydrogen bonding and dipole–dipole interactions [45]. In contrast, anthocyanins and flavonoids show a higher affinity for polyalcohol-based NaDES, primarily due to their milder pH compared to acid-based systems and their high hydrogen-bonding capacity, which favor the preservation of protonated and structurally stable forms. This stabilization enhances extraction efficiency, particularly for compounds with high antioxidant activity [12,16].

Overall, the acid-based NaDESs are more suitable for the extraction of phenolic acids, whereas polyalcohol-based NaDESs preferentially extract anthocyanins and flavonoids, highlighting the critical role of the HBD nature and molar ratio in tailoring NaDES selectivity toward specific phenolic subclasses.

### 3.8. Effect of Independent Variables on the Total Polyphenolic Content

As presented in Table 6, under the experimental conditions considered in the optimization study, the TPC recovered from the AP powder ranged from 2.74 ± 0.33 to 4.15 ± 0.22 mg GAE/g DW.

The outcomes are fitted to a reduced quadratic model, which exhibits an F-value of 47.65, with high statistical significance of the model, according to Table S4 (Supplementary Materials).

The R^2^ (0.9817), together with a non-significant (*p*-value = 0.9474) Lack of Fit, confirms that the selected experimental factors, temperature (A, °C), extraction time (B, min), and water content (C, %), significantly influence the extraction of polyphenols from AP. In addition, the difference between adjusted and predicted R^2^ was lower than 0.2, which shows the high predictive quality and reliability of the model in describing the response variation.

According to Table S4 (Supplementary Materials), temperature (A) and its quadratic term (A^2^), as well as the quadratic term of water content (C^2^), were not statistically significant, as indicated by *p*-values that were greater than 0.05. Similarly, Soukaina et al. [46] reported that, in a Box–Behnken design (BBD) applied for polyphenols extraction from aerial parts of *Mentha pulegium* ssp. using ChCl:G (1:2), the temperature had no significant effect in yield extraction, whereas the water content and extraction time were highly significant, consistent with the high F-values observed for these independent variables (B and C). To evaluate the individual and interactive effect of the factors on the response variable (R1: TPC), an analysis of variance (ANOVA) was performed on the BBD. Based on the high statistical significance of the model and its terms, a second-order polynomial model was selected (Table S4 (Supplementary Materials)).

R1: TPC = 3.430 + 0.058 + 0.325B + 0.393C − 0.117AB − 0.255AC − 0.192BC + 0.037A2 + 0.164B2 + 0.040C2(9)

According to Equation (9), the water content (C) had the most pronounced positive effect on the extraction of TPC, followed by the extraction time (B) and its quadratic term (B^2^). Increasing the water content from 20% (run 12) to 40% (run 15) led to an increase in TPC of 46.43%, as shown in Table S4 (Supplementary Materials), at 40 °C for 45 min. In general, polyphenols extraction from AP has been carried out using ChCl:G at a 1:2 molar ratio, and there are no reports on optimization with ChCl:G at a 1:1 molar ratio. Adding the water up to 30% facilitated polyphenol extraction from AP, as reported by Rashid et al. [16] for ChCl:G (1:2), while Deniz et al. [38] found that 50% water provided the highest TPC values.

In the scientific literature, no studies have reported the optimal water content for the extraction of bioactive compounds using ChCl:G at a 1:1 molar ratio. The higher water content required compared to the value reported by Rashid et al. [16] might be explained by the higher viscosity of the solvent. Huang et al. [40] reported a viscosity of 3.18 Pa·s at 40 °C for ChCl:G (1:1), whereas the ChCl:G (1:2) exhibited a much lower viscosity of 0.13 Pa·s at 40 °C, as reported by Yadav et al. [42]. Thus, adding 40% water reduces viscosity and increases solvent polarity, thereby improving mass transfer and the solubilization capacity of the target compounds from AP, although most studies recommend a maximum of 30% water for the optimal extraction of bioactives. A higher percentage of water leads to a decrease in the yield of bioactive compounds due to the disruption and weakening of molecular interactions between the NaDES components [47,48]. In our study, the addition of 40% water resulted in high TPC values, a trend also reported by Deniz et al. [38], who observed that 50% water in ChCl:G (1:2 molar ratio) maximized TPC recovery from AP.

Prolonging the extraction time facilitated the mass transfer of bioactive compounds from the plant matrix into the solvent, as observed in run 4 and run 5. Maintaining the sample in the ultrasonic bath for 60 min resulted in a polyphenol yield of 3.77 ± 0.55 mg GAE/g DW, representing an increase of 38.69% compared with run 5 performed at 50 °C with a 20% water addition. A longer extraction time allows for the solubilization of polyphenols from the AP and their diffusion into the solvent phase.

The two-way interactions between the independent variables exhibited a negative effect on the polyphenol recovery from AP in the following order: AC > BC > AB. Among these, the AC interaction showed the most pronounced negative effect, confirmed by the highest F-value, according to Table S4 (Supplementary Materials). To better visualize these effects, the 3D and 2D graphical representations are presented in Figure S5 (Supplementary Materials).

The response surface plot (Figure S5 (Supplementary Materials) shows a direct relationship between the response (TPC) and the increase in water content (C) and temperature (A). Water content plays a crucial role in the extraction of polyphenolic compounds, as observed in Figure S5B (Supplementary Materials), followed by extraction time (Figure S5C, Supplementary Materials), whereas temperature alone has a smaller effect. This can be attributed to the specific properties of the solvent, including water content, reduced viscosity, pH, density, and electrical conductivity, as well as the duration of contact between AP and the NaDES throughout ultrasonication [27,29,32]. The experimental conditions that maximized the TPC were 50 °C, 60 min, and 40% water content, yielding a TPC value of 4.15 ± 0.22 mg GAE/g DW. Wang et al. [15] investigated the efficiency of seven different NaDESs in the extraction of total polyphenols (TPC) from AP subjected to blanching at 90 °C for 10 min in a 0.9% NaCl solution, reporting TPC values ranging from 1.80 to 9.97 mg/g AP. The authors showed that ChCl:Oa (1:2 molar ratio, 30% water) and ChCl:Ma (1:1 molar ratio, 30% water) yielded higher TPC values compared to the TPC value obtained in run 18 of Table 6. In contrast, for the other NaDESs, ChCl:1,4 But, ChCl:La, Bet:La, Bet:Suc, and L-Prol:G, the TPC values were lower than the maximum value of 4.15 ± 0.22 mg GAE/g DW obtained in the present study.

### 3.9. Impact of Independent Variables on the Total Flavonoids Content

The experimental conditions (A: temperature; B: extraction time; and C: water content) resulted in a Total Flavonoid Content (R2: TFC, mg QE/g DW) ranging from 0.83 ± 0.26 to 1.06 ± 0.04 mg QE/g DW. The response fit a second-order polynomial model with high statistical significance (*p* < 0.0001) for the F-value. These outcomes indicate that the extraction conditions significantly influenced the TFC, as shown in Table S5 (Supplementary Materials). The non-significant Lack of Fit, confirmed by a *p*-value of 0.1158 and high R^2^, Adj-R^2^, and Pred-R^2^ values exceeding 0.95, indicates the adequacy and predictive quality of the model developed.

ANOVA results showed that water content (C), its interaction with extraction time (BC), and the squared term of time (B^2^) had no significant impact on flavonoid yield, as indicated by their lack of statistical significance (*p* > 0.05). This aspect is evidenced in Table S5 (Supplementary Materials) by the low F-values for these factors compared to the other variables, as well as by the coefficients of the corresponding terms in the regression equation (Equation (10)).

R2: TFC = 0.941 + 0.012A + 0.061B − 0.004C + 0.049AB − 0.028AC – 0.006BC + 0.004A^2^ – 0.018B^2^ – 0.027C^2^ (10)

Table 6 shows that increasing the water content had no significant impact on flavonoid yield, as demonstrated by the comparative analysis between run 4 and run 18, which had identical levels of independent variables A (50 °C) and B (60 min) but were different in C (increasing water from 20% to 40%), resulting in a negligible 1.04% decrease in response.

According to Equation (10), the independent variable exerting the most significant influence on the response yield is extraction time (B), with the highest F-value of 211.93, followed by the interaction between temperature and time (AB), which has an F-value of 69.64. Figure S6 (Supplementary Materials) confirms that prolonging the extraction time at 60 °C and 30% water addition increased the response by 26.19%.

Higher temperature and water addition contributed to a reduction in solvent viscosity and surface tension, while the extended extraction time facilitated the migration of bioactive compounds from the AP under the mechanical effects induced by ultrasonic exposure [38,40]. When optimizing flavonoid extraction from *M. pulegium* using ChCl:G (1:2 molar ratio), Soukaina et al. [46] reported an increase in flavonoid content with the increasing extraction time from 30 min to 90 min, with constant levels for the remaining variables (55 °C; 20% water addition). In this study, run 17 exhibited the highest TFC under experimental conditions of 60 °C, 60 min, and a water content of 30%. According to the literature, based on numerous optimization studies, the optimal extraction temperature for bioactive compounds from various plant matrices using NaDESs does not exceed 60 °C [49]. Increasing temperatures induce physicochemical changes in the solvent, which enhance the solubility of polyphenolic compounds and increase their mass transfer rate from AP cells into the solvent.

### 3.10. Impact of the Independent Variables on the Dependent Variable: Antioxidant Activity

Antioxidant activity values are presented in Table 6, whereas the results of the statistical analysis are shown in Table S6 (Supplementary Materials). It can be observed that the response fit a second-order polynomial model, with an F-value of 102.55. The model explained 99.14% of the experimental variability (R^2^), indicating a very good fit, and the results were significantly influenced by the experimental conditions, confirmed by a non-significant Lack-of-Fit *p*-value of 0.1109.

Following the ANOVA, water content (C), the interaction between extraction time and water content (BC), and the square of the temperature (A^2^), as shown in Table S6 (Supplementary Materials), were found not significant. The antioxidant activity values ranged between 25.74 ± 1.11 and 34.58 ± 0.17 mmol Trolox/g DW. Soukaina et al. [46] reported that the antioxidant activity of the extract obtained with ChCl:G (1:2 molar ratio) was significantly influenced by the water content, the square of the water content, and the square of the temperature, while the remaining factors were not statistically significant. In contrast, in our study, the water content (C) and the square of the temperature (A^2^) were found to be not statistically significant.

R3: DPPH = 29.95 + 2.200A + 0.763B – 0.141C − 0.783AB – 1.420AC + 0.038BC + 0.263A^2^ – 0.579B^2^ + 0.8683C^2^ (11)

According to the regression Equation (11), temperature was the factor that significantly influenced the extraction of bioactive compounds, whereas the interactions between temperature and time (AB) and between temperature and water content (AC) negatively affected the response value. The effect of the interactions between independent variables can also be observed in Figure S7 (Supplementary Materials), providing a clearer understanding of how these factors influence the response value.

At 60 °C and 20% water and keeping the third variable constant (B = 45 min), the extract exhibited the highest antioxidant activity (34.58 ± 0.17 mmol Trolox/g DW). The increase in temperature enhanced the recovery of polyphenolic compounds with antioxidant activity, likely due to the interactions between the solvent components and the AP [46]. Although for TPC and TFC, the water content that facilitated the extraction of bioactive compounds was 40% and 30%, respectively, in the case of antioxidant activity, the lowest level of the variable C (20%) promoted bioactive compound extraction from AP. At a low water content, two effects are observed: (i) the partial disruption of the NaDES supramolecular structure due to the stronger interaction between Cl^−^ and H_2_O compared to Cl^−^ and DLH, and (ii) an increase in the number of hydrogen bonds formed within the solvent [50]. Increasing the water content leads to changes in the physicochemical properties of the solvent, such as reduced density and viscosity, increased pH, decreased surface tension, and altered polarity, which in turn results in the differential extraction of polyphenolic compounds with varying polarities [51].

### 3.11. Maximization of Responses and Validation of Identified Experimental Conditions

The main objective in applying the RSM was to determine the optimal UAE conditions for the recovery of bioactive compounds from AP. As shown in Table 6, each response was maximized under different experimental conditions (temperature, extraction time, and water content). However, the aim of this study was to obtain an extract that simultaneously achieves high values for all three responses (TPC, TFC, and DPPH). According to Figure S8 (Supplementary Materials), the maximization of the responses is located near the apex of the ramp (blue points), while the red points indicate the specific combination of independent variables that lead to maximum responses.

**Figure 4:**
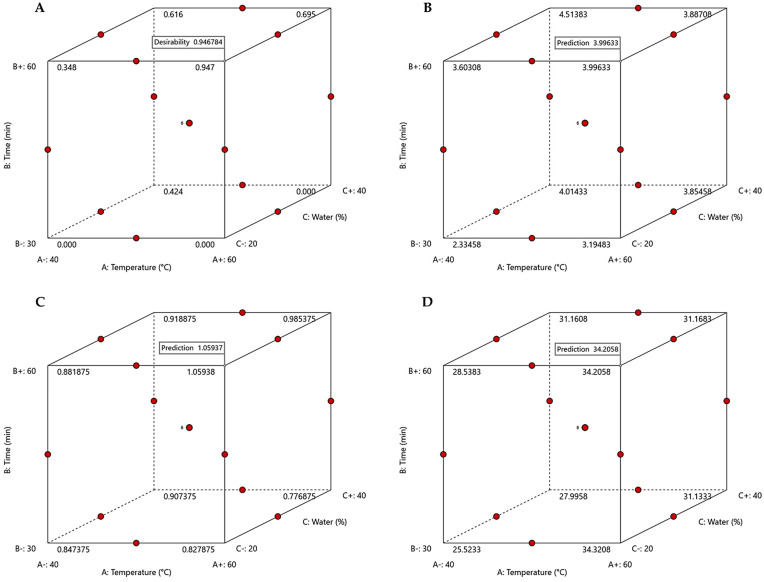
Cube plots of desirability (**A**), TPC (**B**), TFC (**C**), and DPPH (**D**), with predicted values indicated.

The predicted optimal conditions are a temperature of 60 °C, extraction time of 60 min, and 20% water content, as presented in Figure 4, with a desirability of 0.947. To validate these experimental conditions, three replicate extractions were performed at 60 °C for 60 min and 20% water addition, resulting in values of 3.91 ± 0.08 mg GAE/g DW for TPC, 1.05 ± 0.01 mg QE/g DW for TFC, and 34.48 ± 0.25 mmol Trolox/g DW for antioxidant activity.

These responses confirmed the reliability of the optimized solution, as all values fell within the 95% confidence intervals: TPC between 3.78 and 4.23 mg GAE/g DW, TFC between 1.02 and 1.09 mg QE/g DW, and antioxidant activity between 33.45 and 34.96 mmol Trolox/g DW.

### 3.12. Polyphenolic Profile of the Optimized and Validated Extract

Under the validated experimental conditions, the extract was analyzed by HPLC to identify the compounds recovered from AP. The chromatographic profile (Figures S9–S11, Supplementary Materials) consisted of 16 compounds, of which 13.07% were phenolic acids (gallic acid, *p*-coumaric acid, 4-hydroxybenzoic acid, chlorogenic acid, and syringic acid-quantified, with cinnamic and ferulic acids detected in trace amounts), flavan-3-ols (catechin, epigallocatechin, and epicatechin gallate), representing the largest proportion at 82.03%, and anthocyanins, accounting for 2.75% (keracyanin, callistephin, and peonidin-3-*O*-glucoside-quantified, with oenin detected in trace amounts). Flavanones (hesperidin in trace amounts) and a terpenoid (cafestol) contributed 2.15% of the total profile.

The optimized extract obtained using ChCl:G at a 1:1 molar ratio with 20% water at 60 °C for 60 min exhibited a total polyphenol content of 201.06 mg/100 g DW. A comparative analysis with the extract obtained using ChCl:G at a 1:2 molar ratio at 50 °C for 30 min, and with 20% water content, presented in Table 6, revealed notable differences in the extracted amounts and the identified compounds. The validated extract contained significantly higher levels of gallic acid, 4-hydroxybenzoic acid, chlorogenic acid, *p*-coumaric acid, and syringic acid compared to the profile in Table 5. The amounts of catechin and epigallocatechin were substantially increased, whereas epicatechin was not detected, and epicatechin gallate was present in lower contents. The concentration of peonidin-3-O-glucoside remained consistent, while keracyanin was additionally quantified. No flavanols or flavanones were detected in the validated extract. The experimental conditions employed during the validation phase ensured a polyphenolic profile, analyzed by HPLC, slightly lower in total content (201.06 mg/100 g DW) compared to that reported in Table 5 (224.38 mg/100 g DW). However, the amounts of specific phenolic acids, anthocyanins, and certain flavan-3-ols were substantially higher. These findings indicate that the selected solvent significantly influences the recovery efficiency of specific polyphenolic compounds from AP.

Rashid et al. [16] optimized the UAE of lyophilized AP using ChCl:G (1:2 molar ratio) at 40 °C for 40 min, with an S/L ratio of 1:30 g/mL, water content of 30%, acoustic intensity of 83.1 W/cm^2^, and a cycle duty of 75%. Under these conditions, high concentrations of quercetin (142.8 ± 4.08 mg/g of freeze-dried AP), chlorogenic acid (118.10 ± 3.35 mg/g), gallic acid (97.2 mg/g), phloretin (80.4 ± 3.49 mg/100 g), phloridzin (50.1 ± 6.24 mg/100 g), and rutin (21.9 ± 2.64 mg/100 g) were reported. Vlad et al. [52] used freeze-dried AP and reported a complex phenolic profile, comprising phenolic acids, flavan-3-ols, xanthines, flavonols, and flavanones. The authors reported high concentrations of gallic acid (84.42 ± 2.26 mg/g DW AP), caffeic acid (3.62 ± 0.80 mg/g DW), ellagic acid (2.90 ± 0.02 mg/g DW), and protocatechuic acid (2.88 ± 0.02 mg/g DW), which were not detected in the optimized extract analysis. However, higher levels of catechin (95.32 ± 4.09 mg/100 g DW AP) and epigallocatechin (56.30 ± 0 mg/100 g DW AP) were obtained in the optimized extract compared to those reported by Vlad et al. [46].

Four anthocyanins were quantifiable: peonidin-3-*O*-glucoside, keracyanin (cyanidin-3-*O*-galactoside), callistephin (pelargonidin-3-glucoside), and trace amounts of oenin (malvidin-3-*O*-glucoside). These compounds have been quantified in the red apple peel [5] and have also been identified in AP from the Crimson Snow cultivar, with keracyanin (2.11 ± 0 mg/100 g DW AP) being the predominant compound, in good agreement with Chen et al. [53].

The variation in the quantified polyphenol content is influenced by several factors, including the type of apples used for obtaining AP, the applied drying method, CD drying [4,36,37] or freeze drying [16,52], and the UAE conditions, such as temperature, extraction time, and amplitude [16,38,39]. Additionally, the physicochemical characteristics of the NaDESs, such as tunability, added water content, and viscosity, play an important role, as they directly affect the solubility and mass transfer of the target compounds [11,12,39].

According to the obtained values, the concentrations of certain compounds were significantly higher compared to those reported in Table 5. This behavior has been previously explained in the literature by the influence of the HBD proportion in NaDES formulations. Specifically, an increase in the HBD content beyond the optimal molar ratio leads to a reduction in flavonoid extraction efficiency, which can be attributed to the intensified hydrogen bonding interactions occurring between the HBA and the HBD rather than between the solvent and the flavonoid compounds [39].

## 4. Conclusions

The obtained results demonstrate that the drying method significantly influences drying kinetics, color characteristics, and the preservation of polyphenolic compounds with high antioxidant activity in AP. Drying at 70 °C under CA improved polyphenol preservation and resulted in lower browning index values, while IR drying ensured a shorter drying time and reduced specific energy consumption. The polyol-based NaDES system (choline chloride–glycerol, 1:1 molar ratio with 20% water) proved to be the most efficient and suitable option for polyphenolic extraction, attributed to polarity, tunability, and the preservation of flavonoid protonated forms. Optimization using a Box–Behnken design indicated that the conditions of 60 °C, 60 min, and 20% maximize both the polyphenol content and antioxidant activity. The optimized extract was characterized by a high prevalence of flavan-3-ols and phenolic acids. These findings support the potential of neutral NaDESs as an alternative to hydroalcoholic solvents for recovery of polyphenols from AP. Future research should focus on enhancing the practical applicability of NaDES-based extracts.

## Figures and Tables

**Table 1:** D_eff_ and E_a_ values of AP during convective air and infrared drying methods at 50 °C, 60 °C, and 70 °C.

Sample Code	t, °C	D_eff_ × 10^−9^, m^2^/s	R^2^	E_a_, kJ/mol	R^2^
AP_50_CA	50	1.40 ± 0.01 ^c,B^	0.9979	33.71 ± 0.92 ^a^	0.9824
AP_60_CA	60	2.21 ± 0.04 ^b,B^	0.9962		
AP_70_CA	70	2.90 ± 0.09 ^a,B^	0.9902		
AP_50_IR	50	2.14 ± 0.02 ^c,A^	0.9953	23.64 ± 0.92 ^b^	0.9532
AP_60_IR	60	3.07 ± 0.06 ^b,A^	0.9922		
AP_70_IR	70	3.56 ± 0.11 ^a,A^	0.9849		

Note: Different uppercase letters (A,B) indicate significant differences (*p* < 0.05) between powders dried with different drying methods using the same temperatures, while different lowercase letters (a,b,c) indicate significant differences (*p* < 0.05) between D_eff_ values for each drying method, respectively, and E_a_ values between the drying methods, based on Tukey’s test.

**Table 2:** Color parameters of fresh and dried AP during different drying methods used at different temperatures.

Sample Code	L*	a*	b*	∆E	C*	h*	BI	YI	WI
AP_F	37.30 ± 1.01	13.88 ± 0.78	25.25 ± 1.28	-	28.80 ± 1.30	61.20 ± 1.60	25.40 ± 1.60	96.70 ± 2.90	31.00 ± 0.70
AP_50_CA	37.26 ± 1.85 ^C,b^	14.93 ± 0.87 ^A,a^	24.22 ± 0.59 ^B,b^	2.12 ± 0.50 ^C,b^	28.55 ± 0.24 ^C,b^	58.35 ± 2.08 ^B,b^	27.26 ± 2.72 ^A,a^	92.95 ± 2.42 ^A,a^	31.10 ± 1.67 ^C,b^
AP_60_CA	42.93 ± 0.85 ^B,b^	14.19 ± 0.26 ^A,a^	27.34 ± 0.12 ^A,b^	5.12 ± 0.76 ^B,b^	30.81 ± 0.09 ^A,b^	62.57 ± 0.50 ^A,b^	22.73 ± 0.79 ^B,a^	91.00 ± 1.67 ^A,a^	35.15 ± 0.78 ^B,b^
AP_70_CA	48.66 ± 0.74 ^A,b^	13.09 ± 0.25 ^B,b^	27.10 ± 0.27 ^A,b^	11.81 ± 0.94 ^A,b^	30.10 ± 0.30 ^B,b^	64.22 ± 0.40 ^A,b^	18.70 ± 0.44 ^C,a^	79.58 ± 0.79 ^B,a^	40.49 ± 0.56 ^A,b^
AP_50_IR	46.60 ± 1.30 ^C,a^	13.41 ± 0.39 ^A,b^	28.13 ± 0.60 ^B,a^	10.12 ± 0.76 ^B,a^	31.17 ± 0.68 ^B,a^	64.51 ± 0.37 ^A,a^	19.94 ± 0.24 ^A,b^	86.27 ± 1.05 ^A,b^	38.16 ± 0.81 ^C,a^
AP_60_IR	50.11 ± 1.07 ^B,a^	13.53 ± 0.33 ^A,b^	28.86 ± 0.35 ^AB,a^	13.66 ± 1.01 ^A,a^	31.87 ± 0.37 ^AB,a^	64.89 ± 0.54 ^A,a^	18.77 ± 0.57 ^B,b^	82.28 ± 1.20 ^B,b^	40.80 ± 0.79 ^B,a^
AP_70_IR	52.35 ± 0.60 ^A,a^	13.60 ± 0.37 ^A,a^	29.31 ± 0.48 ^A,a^	14.61 ± 0.64 ^A,a^	32.31 ± 0.58 ^A,a^	65.11 ± 0.35 ^A,a^	18.10 ± 0.52 ^B,a^	79.99 ± 1.23 ^C,a^	42.42 ± 0.50 ^A,a^

Note: AP_F—fresh AP, AP_50_CA, AP_60_CA, and AP_70_CA—dried AP powder with CA drying at 50 °C, 60 °C and 70 °C; AP_50_IR, AP_60_IR, and AP_70_IR—dried AP powder with IR drying at 50 °C, 60 °C, and 70 °C; L*—lightness; a*—redness/greenness; b*—yellowness/blueness; ∆E—total color difference; C*—chroma; h*—hue angle; BI—browning index; YI—yellowness index; and WI—whiteness index. Different uppercase letters (A–C) indicate significant differences (*p* < 0.05) between powders dried at different temperatures using the same drying method, while different lowercase letters (a,b) indicate significant differences (*p* < 0.05) between powders dried at the same temperature using different drying methods, based on Tukey’s test.

**Table 3:** Polyphenolic content and antioxidant activity of AP powders dried by convective air and infrared drying methods at 50 °C, 60 °C, and 70 °C.

Sample Code	TPC, mg GAE/g DW	TFC, mg QE/g DW	DPPH, mmol Trolox/g DW
AP_50_CA	7.21 ± 0.14 ^B,a^	1.14 ± 0.10 ^A,a^	20.69 ± 0.62 ^B,a^
AP_60_CA	7.48 ± 0.35 ^A,B,a^	1.00 ± 0.04 ^A,B,a^	21.48 ± 0.23 ^A,B,a^
AP_70_CA	8.03 ± 0.39 ^A,a^	0.91 ± 0.07 ^B,a^	22.13 ± 0.51 ^A,a^
AP_50_IR	6.69 ± 0.28 ^A,b^	0.95 ± 0.03 ^A,b^	20.90 ± 0.51 ^A,a^
AP_60_IR	7.02 ± 0.23 ^A,a^	0.99 ± 0.07 ^A,a^	21.41 ± 0.64 ^A,a^
AP_70_IR	7.18 ± 0.12 ^A,b^	1.01 ± 0.01 ^A,a^	21.53 ± 0.09 ^A,a^

Note: TPC—Total Polyphenolic Content expressed as mg gallic acid equivalent (GAE)/g dry weight (DW) AP powder; TFC—Total Flavonoids Content expressed as mg quercetin equivalent (QE)/g dry weight (DW) AP powder; and DPPH—antioxidant activity expressed as mmol Trolox/g dry weight (DW) AP powder. Different uppercase letters (A,B) indicate significant differences (*p* < 0.05) between powders dried at different temperatures using the same drying method, while different lowercase letters (a,b) indicate significant differences (*p* < 0.05) between powder dried at the same temperature using different drying methods, based on Tukey’s test.

**Table 4:** Conventional and alternative solvents used for the extraction of polyphenolic compounds and antioxidant activity of AP dried at 70 °C using CA drying (AP_70_CA).

Sample Code	Solvent Used for UAE	pH Solvent	TAC, mg C3G/g DW	TPC, mg GAE/g DW	TFC, mg QE/g DW	DPPH, mmol Trolox/g DW
H_2_O	water	6.90 ± 0.15	0.17 ± 0.02 ^B^	5.00 ± 0.06 ^B^	0.75 ± 0.03 ^C^	9.40 ± 0.52 ^E^
EtOH 80%	80% (*v*/*v*) ethanol	5.73 ± 0.30	2.61 ± 1.02 ^A^	6.20 ± 0.09 ^A^	0.86 ± 0.01 ^B^	16.35 ± 0.42 ^C^
ChCl:La (1:1)	Choline chloride: Lactic acid 1:1 molar ratio + 20% water	1.12 ± 0.10	1.91 ± 0.30 ^A,a^	4.15 ± 0.25 ^C,b^	0.57 ± 0.01 ^E,a^	19.91 ± 0.34 ^C,a^
ChCl:La (1:2)	Choline chloride: Lactic acid 1:2 molar ratio + 20% water	0.65 ± 0.21	2.53 ± 0.36 ^A,a^	6.42 ± 0.10 ^A,a^	0.65 ± 0.06 ^D,a^	20.84 ± 0.86 ^C,a^
ChCl:G (1:1)	Choline chloride: Glycerol 1:1 molar ratio + 20% water	2.90 ± 0.12	2.64 ± 0.34 ^A,a^	3.13 ± 0.11 ^D,b^	1.05 ± 0.02 ^A,a^	25.44 ± 0.26 ^A,a^
ChCl:G (1:2)	Choline chloride: Glycerol 1:2 molar ratio + 20% water	3.74 ± 0.28	2.87 ± 0.73 ^A,a^	4.77 ± 0.03 ^B,a^	0.98 ± 0.01 ^A,b^	23.76 ± 0.66 ^B,b^

Note: TAC—total anthocyanin content, expressed as mg cyanidin-3-glicoside (C3G)/g dry weight (DW) AP powder; TPC—Total Polyphenolic Content expressed as mg gallic acid equivalent (GAE)/g dry weight (DW) AP powder; TFC—Total Flavonoids Content expressed as mg quercetin equivalent (QE)/g dry weight (DW) AP powder; and DPPH—antioxidant activity expressed as mmol Trolox/g dry weight (DW) AP powder. Different uppercase letters (A–E) indicate significant differences (*p* < 0.05) between extracts obtained with different solvents for the same variable, while different lowercase letters (a,b) indicate significant differences (*p* < 0.05) between different molar ratios of the same NaDES components, based on Tukey’s test.

**Table 5:** HPLC profile of hydroalcoholic and NaDES extracts.

Compound	EtOH 80%	ChCl:La (1:1)	ChCl:La (1:2)	ChCl:G (1:1)	ChCl:G (1:2)
**Phenolic acid**					
Gallic acid	5.08 ± 0.97 ^a,b^	traces	4.11 ± 0.04 ^b^	5.76 ± 2.23 ^a,b^	7.19 ± 0.13 ^a^
4-Hydroxybenzoic acid	traces	1.06 ± 0.05 ^b^	0.97 ± 0.06 ^b^	1.52 ± 0.13 ^a^	0.33 ± 0.05 ^c^
Protocatechuic acid	n.d.	4.12 ± 0.14 ^b^	n.d.	n.d.	48.44 ± 1.24 ^a^
Vanillic acid	n.d.	traces	n.d.	traces	traces
Ellagic acid	n.d.	9.00 ± 0.07	n.d.	n.d.	n.d.
Syringic acid	n.d.	1.59 ± 0.04 ^b^	1.59 ± 0.03 ^b^	n.d.	1.69 ± 0.09 ^a^
Caffeic acid	n.d.	5.39 ± 0.95 ^a^	2.00 ± 0.09 ^b^	traces	n.d.
*p*-Coumaric acid	n.d.	1.28 ± 0.04	n.d.	n.d.	n.d.
Ferulic acid	n.d.	2.83 ± 0.04	n.d.	traces	n.d.
Sinapic acid	1.34 ± 0.29	n.d.	n.d.	traces	n.d.
Chlorogenic acid	traces	44.77 ± 3.59 ^a^	13.01 ± 0.20 ^b^	12.94 ± 0.18 ^b^	12.73 ± 0.01 ^b^
**Flavan-3-ols**					
Epigallocatechin	65.96 ± 13.56 ^b^	n.d.	185.52 ± 1.62 ^a^	traces	n.d.
Catechin	50.76 ± 2.13 ^c^	49.98 ± 1.67 ^c^	25.29 ± 1.11 ^d^	77.18 ± 31.31 ^b^	98.24 ± 5.93 ^a^
Epicatechin	n.d.	17.10 ± 1.45 ^b^	7.17 ± 2.99 ^c^	85.64 ± 55.04 ^a^	14.07 ± 2.54 ^b,c^
Epicatechin gallate	12.68 ± 1.38 ^b^	14.74 ± 1.38 ^b^	n.d.	47.55 ± 1.02 ^a^	n.d.
**Flavonols**					
Quercetin 3-diglucoside	2.20 ± 0.01	traces	n.d.	n.d.	n.d.
Quercetin 3-glucoside	1.94 ± 0.33	n.d.	n.d.	n.d.	n.d.
Quercetin	1.66 ± 0.05 ^a^	n.d.	3.12 ± 0.02 ^a^	n.d.	n.d.
Quercetin dihydrate	n.d.	3.66 ± 0.88 ^a^	n.d.	3.47 ± 0.90 ^a^	n.d.
Isorhamnetin	n.d.	2.32 ± 0.73 ^a^	n.d.	1.46 ± 0.12 ^b^	n.d.
**Flavones**					
Apigenin	n.d.	2.20 ± 0.03 ^a^	n.d.	2.20 ±0.87 ^a^	n.d.
Luteolin	n.d.	2.79 ± 0.03	n.d.	n.d.	n.d.
**Anthocyanins**					
Peonidin 3-O-glucoside	0.72 ± 0.05 ^b^	n.d.	n.d.	1.49 ± 0.03 ^a^	1.41 ± 0.01 ^a^
Kuromanin (Cyanidin 3-O-glucoside)	traces	n.d.	n.d.	traces	n.d.
**Flavanones**					
Hesperidin	n.d.	n.d.	n.d.	traces	n.d.

Note: n.d. not detected; the values are reported as mean ± standard deviation and expressed as mg/100 g DW of AP. Different lowercase letters (a–d) in the same row indicate significant differences (*p* < 0.05) between solvent extraction use, based on Tukey’s test.

**Table 6:** Experimental plan using a three-level, three-factor Box–Behnken design (BBD) with dependent variables.

Run	A: Temperature, °C	B: Time, min	C: Water, %	R_1_: TPC, mg GAE/g DW	R_2_: TFC, mg QE/g DW	R_3_: DPPH, mmol Trolox/g DW
**1**	50	45	30	3.57 ± 0.69	0.94 ± 0.04	30.19 ± 0.98
**2**	50	30	40	3.90 ± 0.54	0.84 ± 0.06	29.19 ± 0.47
**3**	50	45	30	3.51 ± 0.16	0.95 ± 0.01	29.90 ± 0.82
**4**	50	60	20	3.77 ± 0.55	0.96 ± 0.29	31.22 ± 0.81
**5**	50	30	20	2.74 ± 0.33	0.83 ± 0.26	29.90 ± 1.92
**6**	40	30	30	3.11 ± 0.30	0.89 ± 0.04	25.74 ± 1.11
**7**	60	30	30	3.50 ± 0.66	0.84 ± 0.01	31.88 ± 1.20
**8**	60	45	40	3.69 ± 0.38	0.89 ± 0.00	31.82 ± 0.61
**9**	60	45	20	3.41 ± 0.19	0.96 ± 0.01	34.58 ± 0.17
**10**	40	60	30	4.00 ± 1.09	0.92 ± 0.01	28.96 ± 0.46
**11**	50	45	30	3.36 ± 0.34	0.93 ± 0.09	30.00 ± 1.01
**12**	40	45	20	2.82 ± 0.41	0.90 ± 0.10	27.52 ± 1.09
**13**	50	45	30	3.37 ± 0.29	0.94 ± 0.08	30.12 ± 0.88
**14**	50	45	30	3.47 ± 0.42	0.94 ± 0.05	29.74 ± 0.27
**15**	40	45	40	4.12 ± 0.58	0.94 ± 0.05	30.42 ± 0.15
**16**	50	45	30	3.33 ± 0.25	0.94 ± 0.08	29.77 ± 0.70
**17**	60	60	30	3.92 ± 0.36	1.06 ± 0.04	31.97 ± 0.89
**18**	50	60	40	4.15 ± 0.22	0.95 ± 0.03	30.66 ± 0.12

Note: Data are expressed as mean ± standard deviation; TPC—Total Polyphenolic Content, expressed as mg gallic acid equivalent (GAE)/g dry weight (DW); and TFC—Total Flavonoid Content, expressed as mg quercetin equivalent (QE)/ g dry weight (DW).

## Data Availability

The original contributions presented in this study are included in the article/Supplementary Materials. Further inquiries can be directed to the corresponding author.

## Supplementary Materials

The following supporting information can be downloaded at: https://www.mdpi.com/article/10.3390/antiox15040429/s1.
