# Supplementary material for: Apple Pomace as a Source of Valuable Phenolics: From Drying Kinetics to Optimization of Ultrasound-Assisted Extraction Using Conventional and Alternative Solvents
Source: Antioxidants (Basel). 2026 Mar 29;15(4):429. doi: 10.3390/antiox15040429 (PMC13113185; doi:10.3390/antiox15040429)
Supplement: Supplementary file 1 [file antioxidants-15-00429-s001.zip › antioxidants-4177610-supplementary.pdf]

Table S1. The mathematical models applied for drying of AP.

MR = exp(-kt)	Lewis [18]
MR = exp(-ktⁿ)	Page [19]
MR = aexp(-kt)	Henderson & Pabis [20]
MR = aexp(-kt)+c	Logarithmic - Yaldiz et al. [21]
MR = 1 + at + bt²	Wang & Singh [22]
MR = aexp(-ktⁿ) + bt	Midilli et al. [23]
MR = aexp(-kt)+(1-a)exp(-kbt)	Diffusion Approach [24]

Table S2. Statistical analysis using different mathematical models on experimental data obtained from drying apple pomace (AP).

Mathematical model	Drying method	T, °C	Constants					R^2	RMSE	χ^2
			k	n	a	b	c			
**Lewis/ Newtown [18]**	CA	50	0.0126	-	-	-	-	0.9994	0.68×10^{-2}	4.90×10^{-5}
		60	0.0197	-	-	-	-	0.9966	1.62×10^{-2}	2.82×10^{-4}
		70	0.0231	-	-	-	-	0.9935	2.35×10^{-2}	5.98×10^{-4}
	IR	50	0.0181	-	-	-	-	0.9938	2.29×10^{-2}	5.66×10^{-4}
		60	0.0221	-	-	-	-	0.9913	2.90×10^{-2}	9.22×10^{-4}
		70	0.0264	-	-	-	-	0.9874	3.68×10^{-2}	1.53×10^{-3}
**Page [19]**	**CA**	**50**	**0.0097**	**1.054**	**-**	**-**	**-**	**0.9990**	$\mathbf{8.24 \times 10^{-3}}$	$\mathbf{7.58 \times 10^{-5}}$
		**60**	**0.0094**	**1.172**	**-**	**-**	**-**	**0.9995**	$\mathbf{5.96 \times 10^{-3}}$	$\mathbf{4.10 \times 10^{-5}}$
		**70**	**0.0075**	**1.275**	**-**	**-**	**-**	**0.9994**	$\mathbf{6.96 \times 10^{-3}}$	$\mathbf{5.73 \times 10^{-5}}$
	**IR**	**50**	**0.0065**	**1.238**	**-**	**-**	**-**	**0.9994**	$\mathbf{6.86 \times 10^{-3}}$	$\mathbf{5.52 \times 10^{-5}}$
		**60**	**0.0061**	**1.316**	**-**	**-**	**-**	**0.9997**	$\mathbf{5.77 \times 10^{-3}}$	$\mathbf{4.07 \times 10^{-5}}$
		**70**	**0.0045**	**1.455**	**-**	**-**	**-**	**0.9997**	$\mathbf{5.74 \times 10^{-3}}$	$\mathbf{4.23 \times 10^{-5}}$
**Henderson and Pabis [20]**	CA	50	0.0127	-	1.008	-	-	0.9994	0.65×10^{-2}	4.72×10^{-5}
		60	0.0199	-	1.017	-	-	0.9969	1.55×10^{-2}	2.77×10^{-4}
		70	0.0235	-	1.019	-	-	0.9938	2.28×10^{-2}	6.15×10^{-4}
	IR	50	0.0185	-	1.026	-	-	0.9945	2.17×10^{-2}	5.50×10^{-4}
		60	0.0225	-	1.024	-	-	0.9919	2.80×10^{-2}	9.55×10^{-4}
		70	0.0268	-	1.022	-	-	0.9880	3.61×10^{-2}	1.67×10^{-3}
**Logarithmic [21]**	CA	50	0.0125	-	1.011	-	-0.0064	0.9997	0.49×10^{-2}	2.87×10^{-5}
		60	0.0195	-	1.023	-	-0.0080	0.9974	1.43×10^{-2}	2.57×10^{-4}
		70	0.0227	-	1.029	-	-0.0119	0.9947	2.12×10^{-2}	5.84×10^{-4}
	IR	50	0.0179	-	1.035	-	-0.0125	0.9954	1.20×10^{-2}	5.04×10^{-4}
		60	0.0214	-	1.038	-	-0.0174	0.9933	2.53×10^{-2}	8.83×10^{-4}
		70	0.0253	-	1.041	-	-0.0218	0.9899	3.31×10^{-2}	1.64×10^{-3}
**Wang and Singh [22]**	CA	50	-	-	-0.006	0.85×10^{-5}	-	0.8387	1.09×10^{-1}	1.32×10^{-2}
		60	-	-	-0.008	1.50×10^{-5}	-	0.7691	1.14×10^{-1}	2.07×10^{-2}
		70	-	-	-0.010	2.04×10^{-5}	-	0.7837	1.35×10^{-1}	2.15×10^{-2}
	IR	50	-	-	-0.009	1.65×10^{-5}	-	0.8516	1.12×10^{-1}	1.47×10^{-2}
		60	-	-	-0.011	2.69×10^{-5}	-	0.8911	1.03×10^{-1}	1.29×10^{-2}
		70	-	-	-0.014	4.09×10^{-5}	-	0.9141	0.96×10^{-1}	1.19×10^{-2}

Approximation of diffusion [24]	CA	50	0.0125	-	0.567	1.000	-	0.9985	1.07×10^{-2}	1.37×10^{-4}
		60	0.0196	-	0.556	1.008	-	0.9963	1.71×10^{-2}	3.65×10^{-4}
		70	0.0227	-	0.619	1.000	-	0.9893	2.93×10^{-2}	1.12×10^{-4}
	IR	50	0.0179	-	0.564	0.994	-	0.9921	2.62×10^{-2}	8.71×10^{-4}
		60	0.0219	-	0.548	1.000	-	0.9908	2.92×10^{-2}	1.17×10^{-3}
		70	0.0265	-	0.585	1.009	-	0.9900	3.27×10^{-2}	1.60×10^{-4}
**Midilli et al. [23]**	**CA**	**50**	**0.0100**	**1.049**	**0.998**	**-5.30×10^{-6}**	**-**	**0.9998**	**3.32×10^{-3}**	**1.40×10^{-5}**
		**60**	**0.0089**	**1.190**	**1.000**	**1.22×10^{-6}**	**-**	**0.9999**	**2.05×10^{-3}**	**5.74×10^{-6}**
		**70**	**0.0068**	**1.304**	**0.999**	**0.16×10^{-6}**	**-**	**0.9998**	**3.18×10^{-3}**	**1.46×10^{-5}**
	**IR**	**50**	**0.0062**	**1.255**	**0.999**	**2.90×10^{-6}**	**-**	**0.9999**	**2.73×10^{-3}**	**1.05×10^{-5}**
		**60**	**0.0055**	**1.345**	**1.000**	**2.76×10^{-6}**	**-**	**0.9999**	**1.13×10^{-3}**	**1.99×10^{-6}**
		**70**	**0.0039**	**1.505**	**1.000**	**8.57×10^{-6}**	**-**	**0.9998**	**4.74×10^{-3}**	**4.04×10^{-5}**

Table S3. Explained variance (%) of the most important principal components (PC).

	PC1	PC2	PC3	PC4	PC5	PC6	PC7	PC8
**Eigenvalue**	10.598	7.578	4.277	3.249	0.120	0.089	0.068	0.011
**Proportion**	40.80	29.10	16.50	12.50	0.50	0.30	0.30	0.00
**Cumulative**	40.80	69.90	86.40	98.90	99.3	99.70	99.90	100

Table S4. Analysis of variance (ANOVA) and statistical analysis of the reduced quadratic model for first variable dependent (R1: TPC, mg GAE/g DW).

Source	Sum of Squares	df	Mean Square	F-value	*p*-value
**Model**	2.71	9	0.3017	47.65	< 0.0001
**A-Temperature**	0.0273	1	0.0273	4.31	0.0717
**B-Timp**	0.8463	1	0.8463	133.69	< 0.0001
**C-Water**	1.23	1	1.23	194.81	< 0.0001
**AB**	0.0545	1	0.0545	8.61	0.0189
**AC**	0.2601	1	0.2601	41.09	0.0002
**BC**	0.1478	1	0.1478	23.35	0.0013
**A^2**	0.0058	1	0.0058	0.9225	0.3649
**B^2**	0.1178	1	0.1178	18.61	0.0026
**C^2**	0.0070	1	0.0070	1.11	0.3234
**Residual**	0.0506	8	0.0063		
**Lack of Fit**	0.0033	3	0.0011	0.1152	0.9474
**Pure Error**	0.0474	5	0.0095		
**Cor Total**	2.77	17			
**R^2**	0.9817				
**Adjusted R^2**	0.9611				
**Predicted R^2**	0.9564				

Table S5. Analysis of variance (ANOVA) and statistical analysis of the reduced quadratic model for second variable dependent (R2: TFC, mg QE/g DW).

Source	Sum of Squares	df	Mean Square	F-value	*p*-value
**Model**	0.0486	9	0.0054	38.76	< 0.0001
**A-Temperature**	0.0011	1	0.0011	7.93	0.0226
**B-Time**	0.0295	1	0.0295	211.93	< 0.0001
**C-Water**	0.0001	1	0.0001	0.7035	0.4260
**AB**	0.0097	1	0.0097	69.64	< 0.0001
**AC**	0.0031	1	0.0031	22.11	0.0015
**BC**	0.0001	1	0.0001	0.9493	0.3584
**A^2**	0.0001	1	0.0001	0.5995	0.4610
**B^2**	0.0014	1	0.0014	10.29	0.0125
**C^2**	0.0031	1	0.0031	22.20	0.0015
**Residual**	0.0011	8	0.0001		
**Lack of Fit**	0.0007	3	0.0002	3.30	0.1158
**Pure Error**	0.0004	5	0.0001		
**Cor Total**	0.0497	17			
**R^2**	0.9776				
**Adjusted R^2**	0.9524				
**Predicted R^2**	0.9508				

Table S6. Analysis of variance (ANOVA) and statistical analysis of the reduced quadratic model for third variable dependent (R3: DPPH, mmol Trolox/g DW).

Source	Sum of Squares	df	Mean Square	F-value	*p*-value
**Model**	58.81	9	6.53	102.55	< 0.0001
**A-Temperature**	38.76	1	38.76	608.35	< 0.0001
**B-Timp**	4.65	1	4.65	73.00	< 0.0001
**C-Water**	0.1596	1	0.1596	2.50	0.1521
**AB**	2.45	1	2.45	38.44	0.0003
**AC**	8.01	1	8.01	125.69	< 0.0001
**BC**	0.0056	1	0.0056	0.0883	0.7739
**A^2**	0.3026	1	0.3026	4.75	0.0609
**B^2**	1.46	1	1.46	22.97	0.0014
**C^2**	3.29	1	3.29	51.64	< 0.0001
**Residual**	0.5098	8	0.0637		
**Lack of Fit**	0.3418	3	0.1139	3.39	0.1109
**Pure Error**	0.1679	5	0.0336		
**Cor Total**	59.32	17			
**R^2**	0.9914				
**Adjusted R^2**	0.9817				
**Predicted R^2**	0.9037				

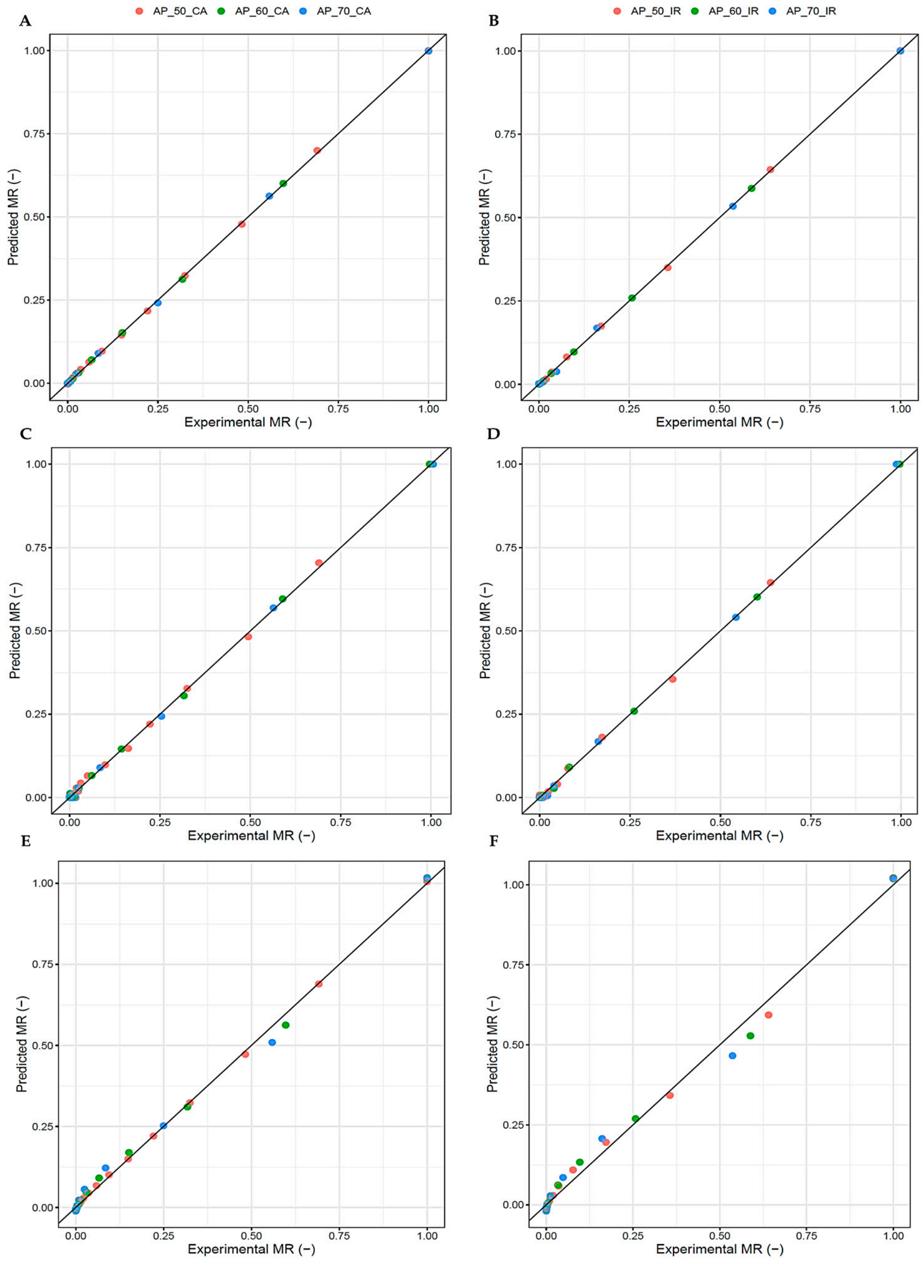

Figure S1. Predicted MR versus experimental MR of AP for both drying methods, fitted using the Midilli (**A-B**), Page (**C-D**) and Logarithmic (**E-F**) models.

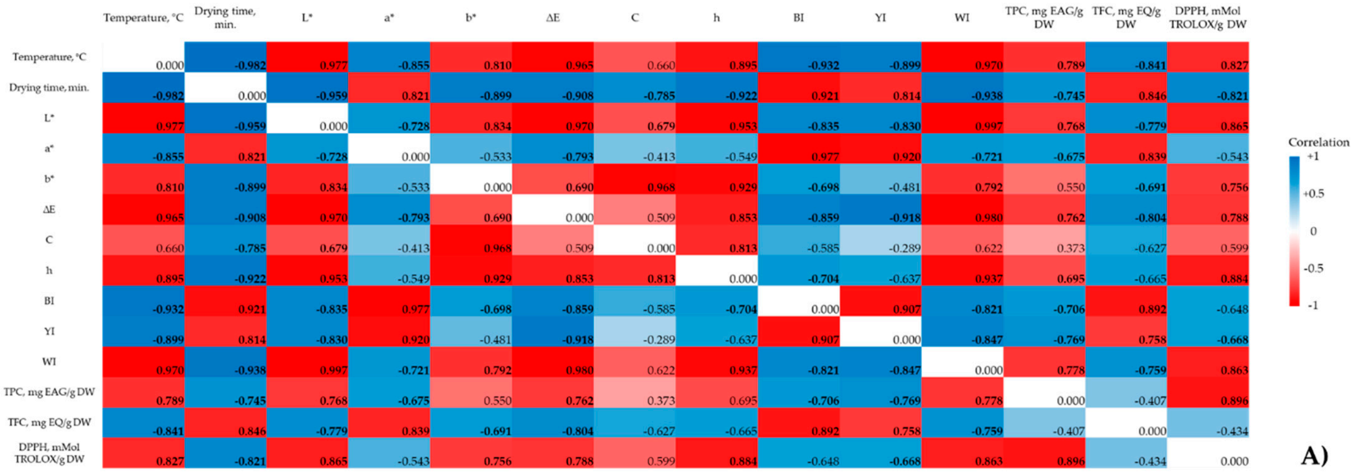

A)

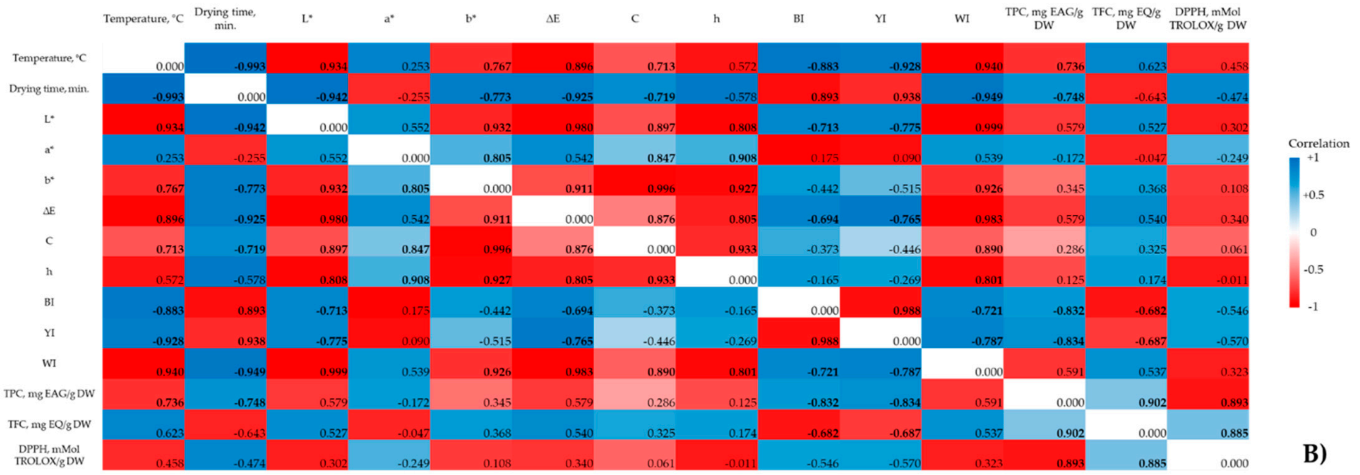

B)

Figure S2. Pearson correlation coefficients (*r*) for the independent and dependent variables analyzed for AP powder drying by CA (A) and by IR (B). Bold number indicates statistically significance correlation (*p*-value < 0.05). Red represents positive correlation, while blue represents negative correlation.

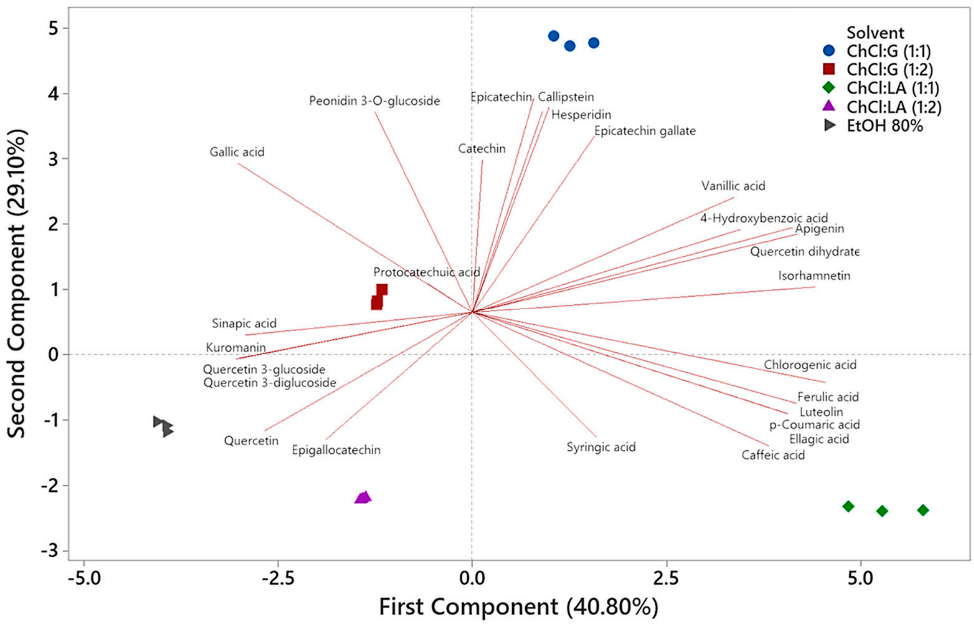

Figure S3. Multivariate plot (PCA) of 27 polyphenolic compounds identified by HPLC analysis from hydroalcoholic and NaDES extracts, based on the first two principal components.

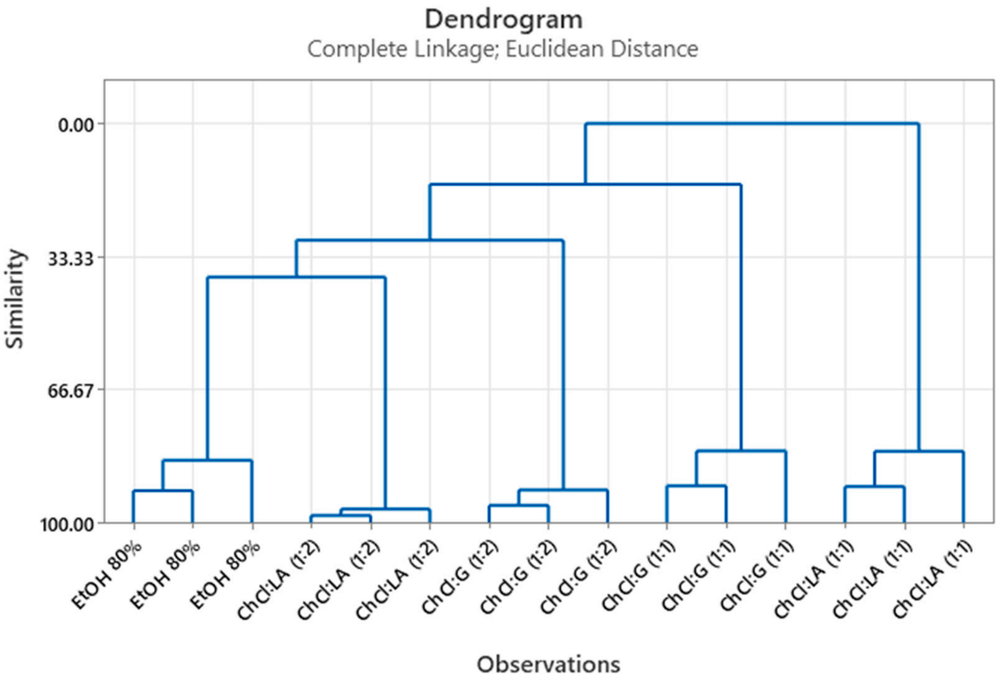

Figure S4. Dendrogram of hierarchical cluster analysis (HCA) based on the HPLC profile of hydroalcoholic and NaDES extracts.

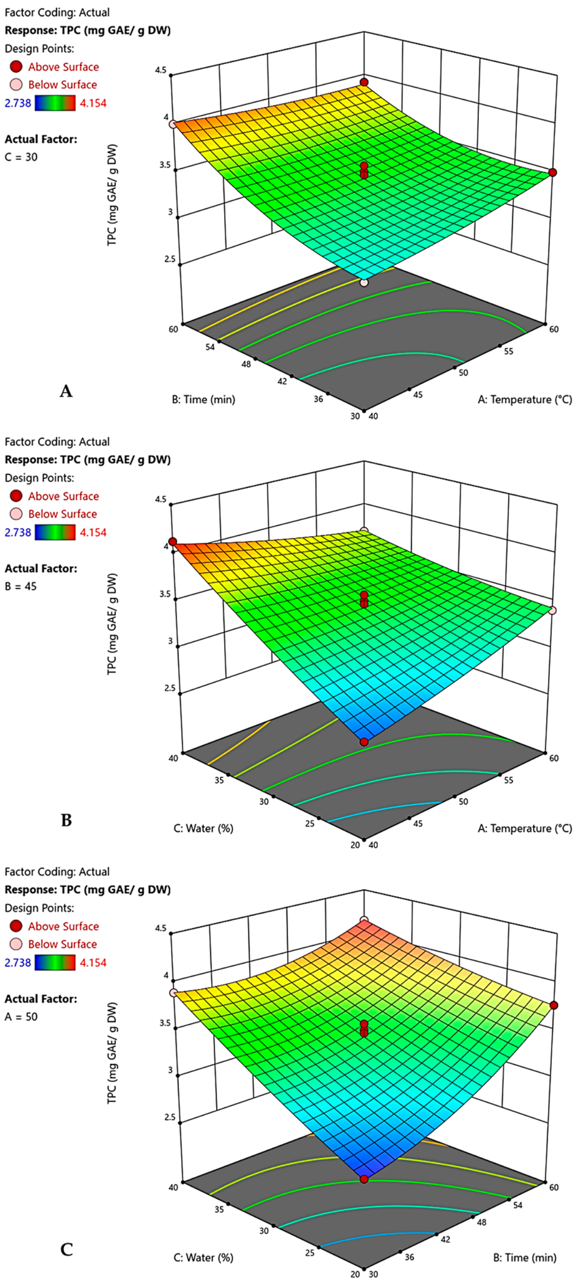

Figure S5. Tree-dimensional (3D) and two-dimensional (2D) graphical representation of interaction effect between two factors while the third is held at a constant value, for the first response (R1: TPC, mg GAE/ g DW): A is interaction between temperature (A) and time (B), B is interaction between temperature (A) and water (C) and C is interaction between time (B) and water (C).

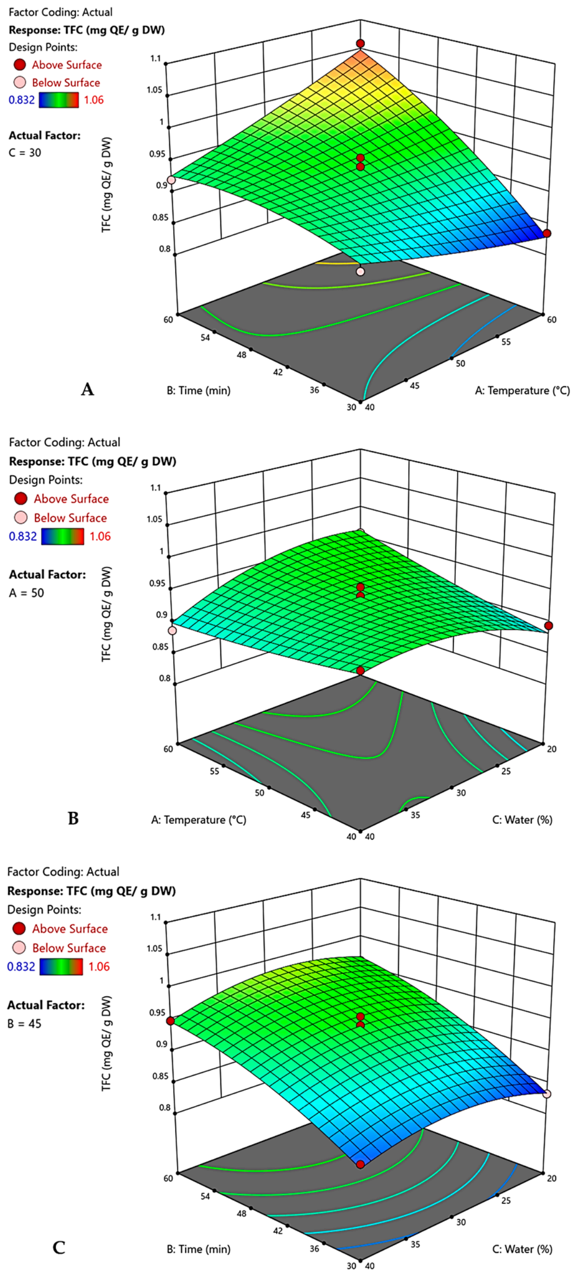

Figure S6. Tree-dimensional (3D) and two-dimensional (2D) graphical representation of interaction effect between two factors while the third is held at a constant value, for the second response (R2: TFC, mg QE/g DW): A is interaction between temperature (A) and time (B), B is interaction between temperature (A) and water (C) and C is interaction between time (B) and water (C).

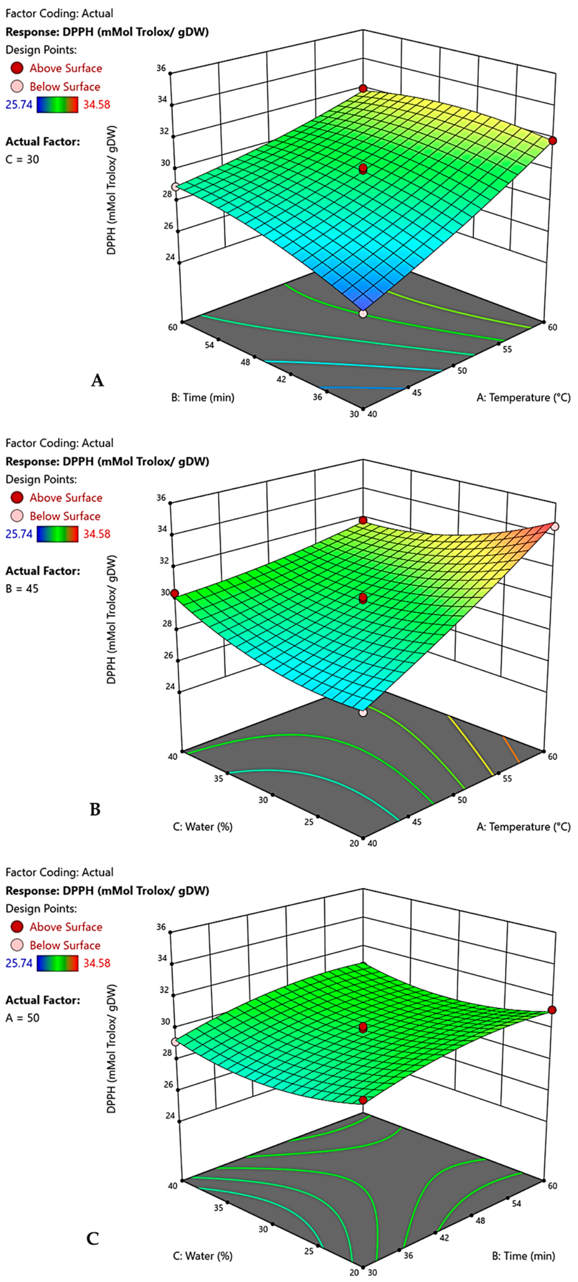

Figure S7. Tree-dimensional (3D) and two-dimensional (2D) graphical representation of interaction effect between two factors while the third is held at a constant value, for the third response (R3: DPPH, mmol Trolox/g DW): A is interaction between temperature (A) and time (B), B is interaction between temperature (A) and water (C) and C is interaction between time (B) and water (C).

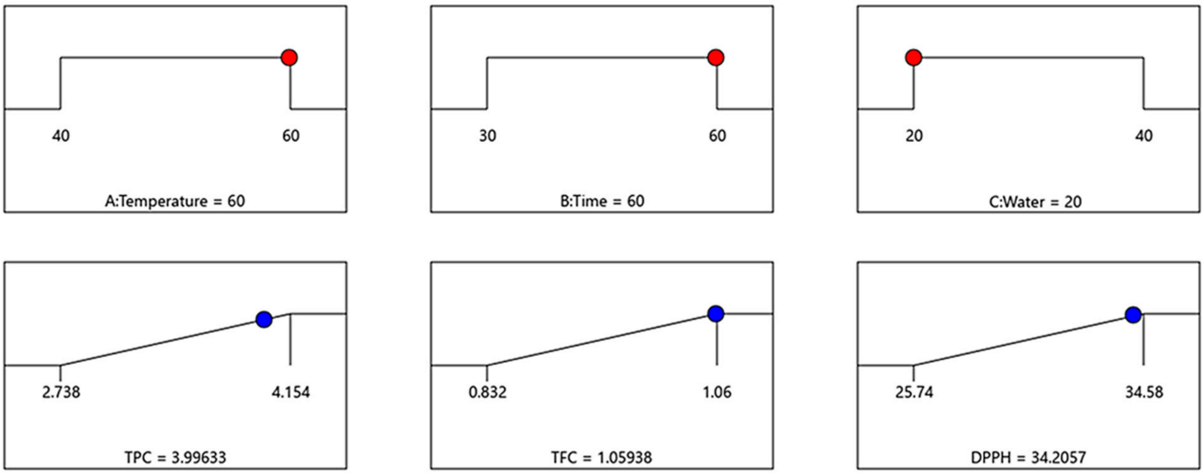

Figure S8. Ramp plot for the optimized response, where the red circles indicate the value of the independent variable (A, B and C) necessary to obtain the maximum response (TPC, TFC and DPPH) indicate with the blue circles.

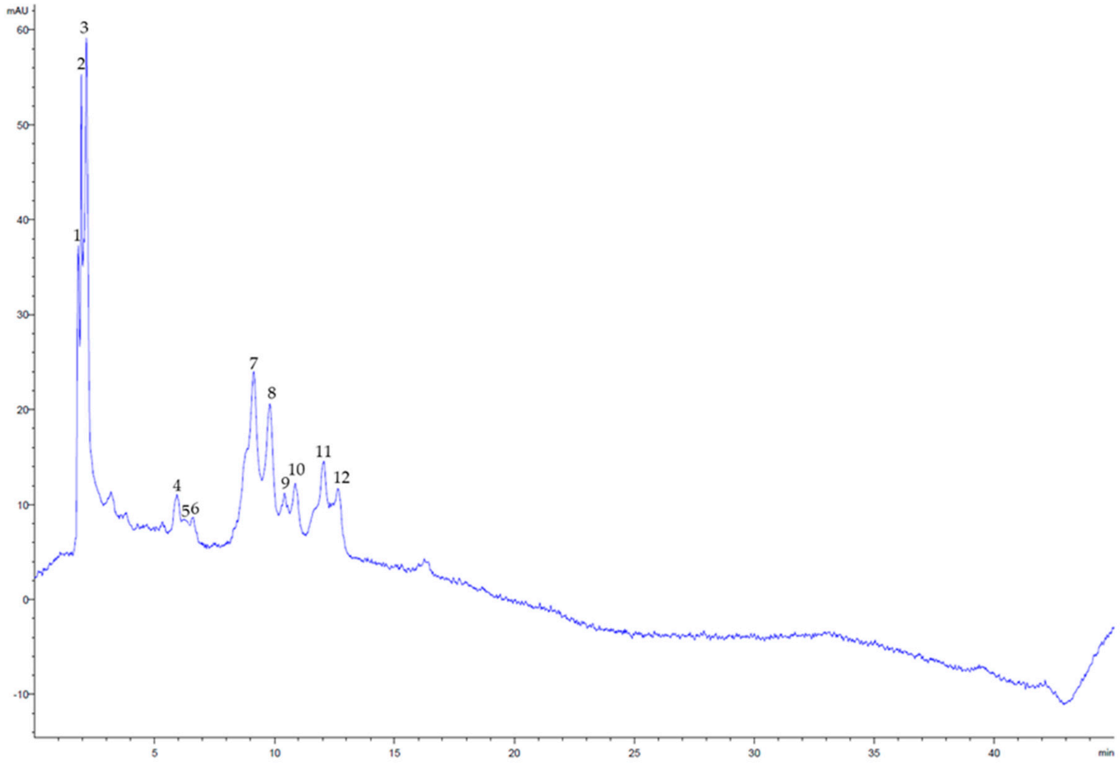

Figure S9. Polyphenolic profile of optimized extracts at 280 nm: 1 – epigallocatechin, 2 – catechin, 3, 4 – unidentified compounds, 5 – 4-hydroxybenzoic acid, 6 – chlorogenic acid, 7 – syringic acid, 8 – caffeine, 9 - epicatechin gallate, 10 – *p*-coumaric acid

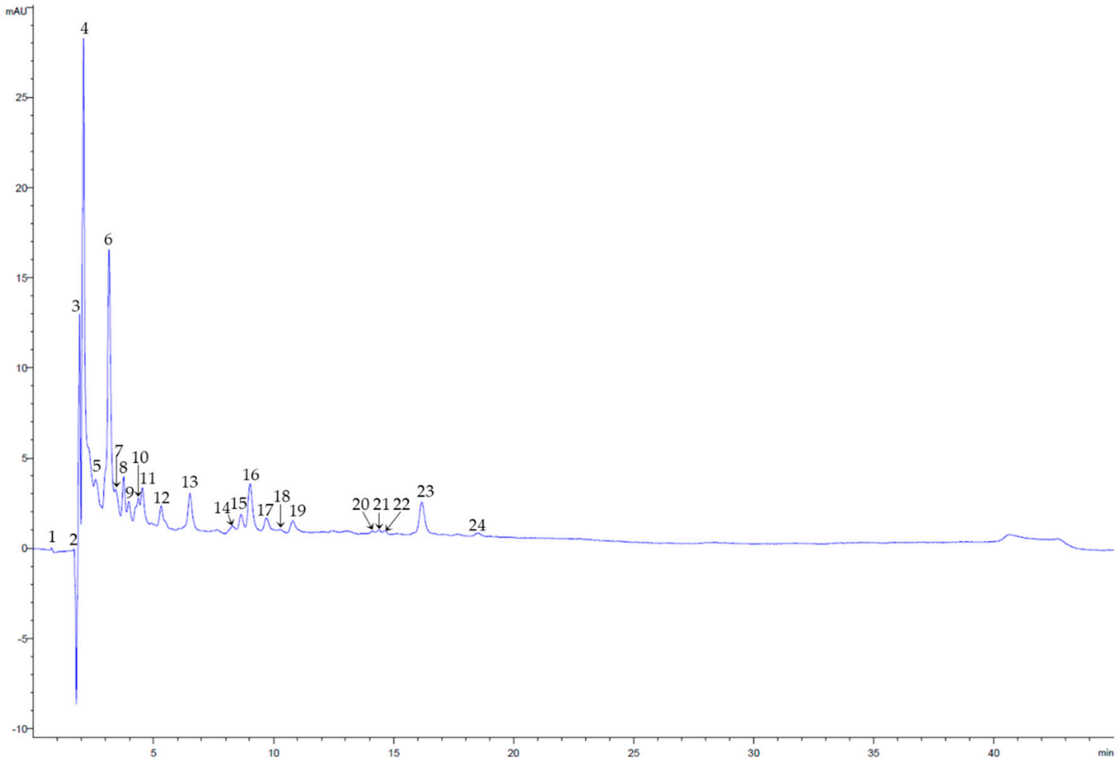

Figure S10. Polyphenolic profile of optimized extracts at 320 nm: 1, 2 – unidentified compound, 3 – gallic acid, 4 – catechin, 5 – unidentified compound, 6 – 4-hydroxybenzoic acid, 7 – unidentified compound, 8 – chlorogenic acid, 9-13 – unidentified compounds, 14 – ferulic acid, 20 – oenin (malvidin-3-*O*-glucoside), 21 - – unidentified compound, 22 – hesperidin, 23 – cinnamic acid, 24 - unidentified compound.

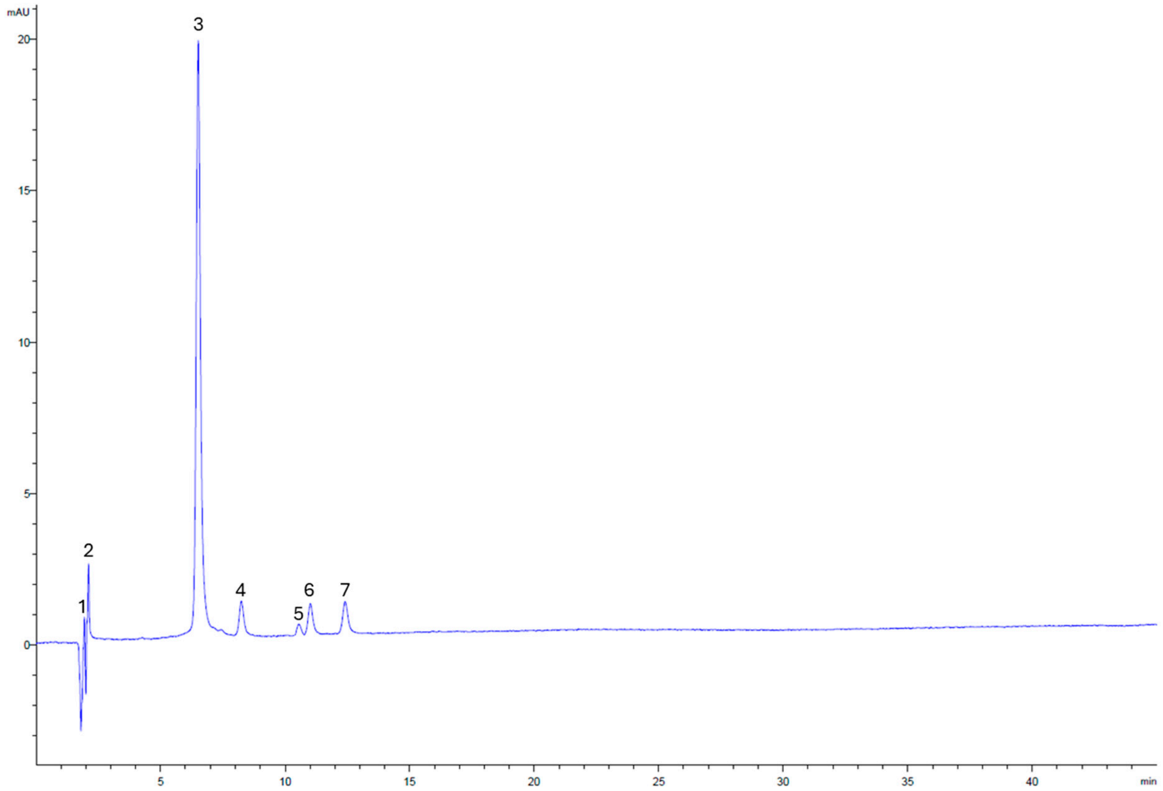

Figure S11. Polyphenolic profile of optimized extracts at 520 nm: 1 – gallic acid, 2 – catechin, 3-4 – unidentified compounds, 5 – keracyanin (cyanidin-3-*O*-galactoside), 6 – callipsthein (pelargonidin-3-glucoside), 7 – peonidin-3-O-glucoside
